# Quebec lung, liver and heart transplant recipients’ perspectives on self-narratives and their experiences in creative writing workshops during the transplantation journey: a qualitative study

**DOI:** 10.1136/bmjopen-2024-096791

**Published:** 2026-07-03

**Authors:** Laurence Ducharme, Marie-Françoise Malo, Aliya Affdal, Fabian Ballesteros, Laurence Laneuville, Leonore Brassard, Benjamin Gagnon-Chainey, Pascale Millot, Catherine Mavrikakis, Simon Harel, Marie-Chantal Fortin

**Affiliations:** 1 Université de Montréal Faculté de Médecine, Montreal, Quebec, Canada; 2 École de santé publique, Bioethics Department, Université de Montréal, Montreal, Quebec, Canada; 3 Centre Hospitalier de l’Université de Montréal, Montreal, Quebec, Canada; 4 Département de psychiatrie et d’addictologie, Université de Montréal Faculté de Médecine, Montreal, Quebec, Canada; 5 Chaire McConnell-Université de Montréal sur les récits de don et de la vie en contexte de soins, Université de Montréal, Montreal, Quebec, Canada; 6 Département de lettres et communication sociale, Université du Québec à Trois-Rivières, Trois-Rivières, Quebec, Canada; 7 Département des littératures de langues françaises, Université de Montréal Faculté des Arts et des Sciences, Montreal, Quebec, Canada

**Keywords:** TRANSPLANT MEDICINE, QUALITATIVE RESEARCH, Peer Group, Literature

## Abstract

**Background:**

Although solid organ transplantation is a life-saving procedure, it is also associated with numerous challenges for patients. Creative writing has been described as a therapeutic tool for patients with chronic disease. Between 2020 and 2022, we held creative writing workshops with patients and professional writers. During these workshops, patients were invited to explore different aspects of their transplantation journey through different literary styles.

**Objectives:**

The aim of this study is to understand lung, liver and heart transplant patients’ perspectives on the role of patient self-narratives and creative writing workshops during their transplantation journey, and collect testimonies on their experiences in the workshops.

**Design:**

Focus groups and individual interviews.

**Setting:**

The Centre hospitalier de l’Université de Montréal (CHUM) lung and liver transplant programmes and the Montreal Heart Institute (MHI) heart transplant programme.

**Participants:**

Lung, liver and heart transplant recipients attending the CHUM and the MHI transplant clinic between September 2020 and August 2022.

**Methods:**

Before the creative writing workshop, we conducted two focus groups and individual interviews with 22 lung recipients, five liver transplant recipients and four heart transplant patients. After their participation in the creative workshop, we conducted 17 semi-structured interviews with nine lung transplant recipients, four liver transplant recipients and one heart transplant recipient (three participants participated twice in interviews). Interviews were transcribed for subsequent qualitative description analyses.

**Results:**

Most participants had previous experiences of sharing their transplant stories and considered that a web-based platform where transplant patients share their stories could be highly beneficial for future transplant patients and the public. The creative workshops were described as ‘therapeutic’ since they let participants express their emotions and realities. Peer support and connection to other people contributed to normalise their transplant journey and helped participants put their experiences into perspective.

**Conclusions:**

Creative writing workshops and peer support through the sharing of self-narratives are a feasible approach that participants have described as beneficial. Future studies are needed to assess the benefits of patients’ creative writings on other patients and on caregivers.

**STRENGTHS AND LIMITATIONS OF THIS STUDY**

- The qualitative design of our study allowed us to gain in-depth insights into participants’ perspectives on self-narratives and their experiences in creative writing workshops.
- Including transplant recipients of different organs enriched our understanding of how self-narratives and creative writing workshops may support diverse patient experiences.
- Using both individual interviews and focus groups generated richer thematic content and enhanced the depth of our analysis.
- One limitation of our study is that most participants were White, highly educated and recruited from three urban transplant programmes, which may limit the generalisability of our findings.

## Introduction

Heart, lung and liver transplantations are treatments of choice for end-stage cardiac, lung or liver disease.^1^ Living with a life-threatening illness leads to personal and social changes that can be difficult to cope with.^2 3^ Despite reporting a better quality of life after transplantation,^4^ emotional and psychological impacts of a transplant can remain long after the physical improvement of the recipient.^5 6^

Storytelling and art-based intervention (ABI) have gained a lot of traction in recent years as ways to bolster patients’ recovery journey.^7^ These approaches have been shown to benefit patients with mental illnesses,^8 9^ patients with cancer^10^ and patients with chronic illnesses.^11 12^ ABIs can encompass several different art forms—from creative writing to visual arts to music.^8^ Storytelling can help patients make sense of their emotions and can offer a setting and a framework for them to explore difficult aspects of their illness journey.^13^ The subsequent dissemination of these stories may also help others during their own journey of care towards and after transplantation.^14^ Our research group has already demonstrated, in previous work, the benefits of creative writing workshops and the diffusion of these self-narratives and creative writings for kidney transplant recipients, kidney transplant candidates and living kidney donors.^15^ This study expands on our earlier research by examining the perspectives of heart, lung and liver transplant recipients on the role of creative writing and their experiences participating in creative writing workshops.

The objective of this study was twofold. First, prior to the creative writing workshops, transplant patients were invited to share their perspectives on the experience of narrating their transplantation journey. Second, patients who participated in the creative writing workshops were asked about their experiences in the workshops and the impact of sharing their self-narratives and creative writings. This study aims to deepen understanding of the role that creative writing workshops and ABI can play in supporting lung, liver and heart transplant recipients throughout their transplantation journey.

## Methods

This study was exploratory in nature and used semistructured interviews and focus groups with heart, lung and liver transplant recipients. It employed the Consolidated criteria for reporting qualitative research (COREQ) checklist for reporting qualitative research.^16^ We conducted individual interviews and focus groups before participants attended the creative writing workshops, and individual interviews after the workshops. All recipients received organs from deceased donors, recovered with valid authorisation and in accordance with Canadian legislation, international standards and the ethical principles outlined in the Declaration of Istanbul on Organ Trafficking and Transplant Tourism.^17^ After the workshops, there was a separate consent process regarding the decision to post the creative writings on the website anonymously or not.

### Interviews and focus groups conducted before the creative writing workshops

Recruitment for focus groups and interviews was carried out between December 2020 and August 2022. Convenience sampling was the chosen approach for recruiting participants. French-speaking adults who were recipients of a heart, lung or liver transplant, and were followed at the two transplant clinics, were invited to participate by means of letters, posters, emails and flyers distributed at the transplant clinic. Directors of the lung and liver transplant programmes sent an invitation email to 377 patients (358 lung recipients and 19 liver recipients), followed by a telephone call made by a member of the research team (FB or M-FM). The heart transplant clinic approached the patients directly and obtained their informed consent before referring them to the research coordinator (FB).

**Figure 1:**
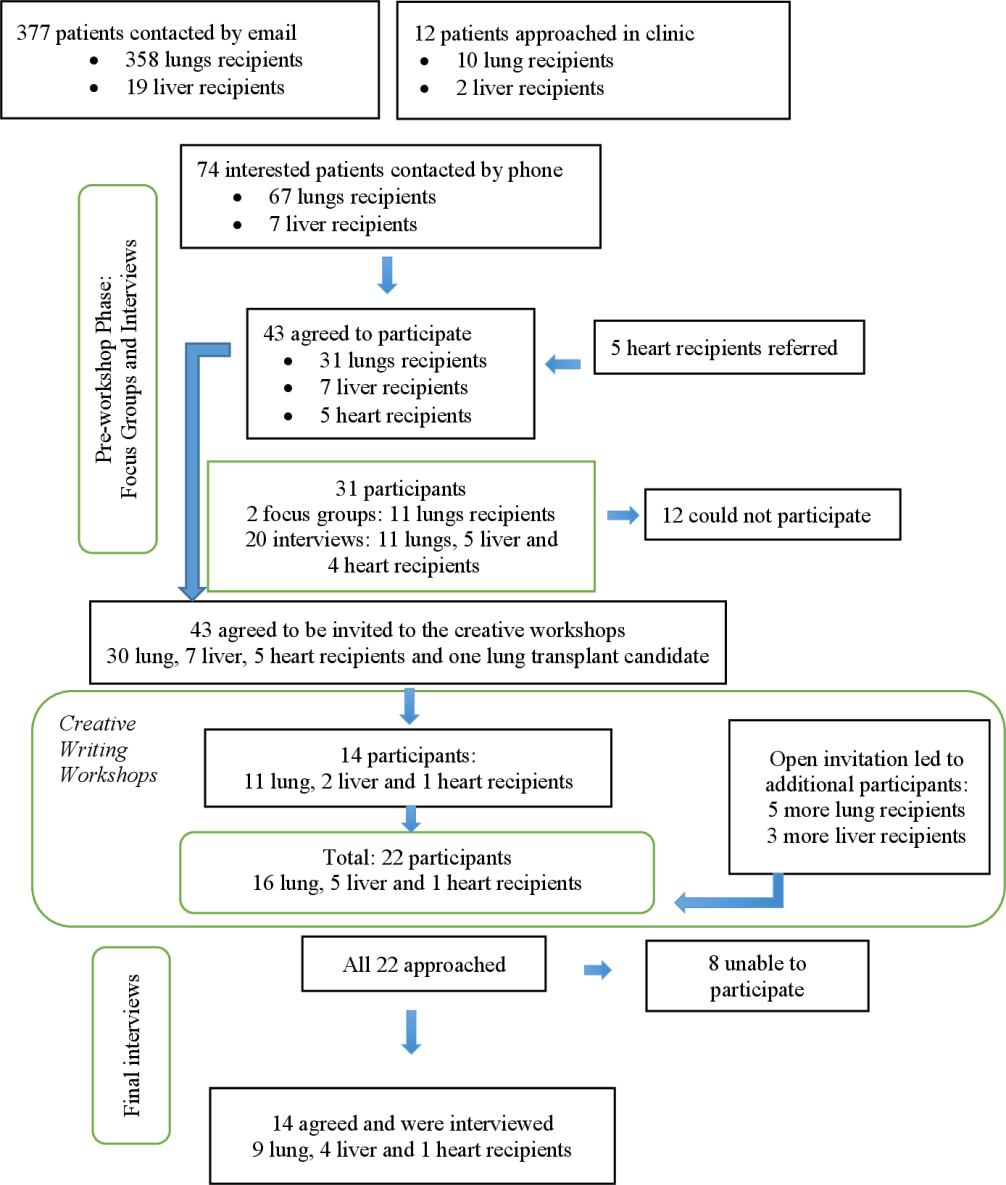
The concept of the study. Combining carbon-ion radiotherapy with atezolizumab plus bevacizumab may provide synergistic therapeutic benefit by activating immune responses while suppressing pro-tumourigenic signalling pathways.

67 lung recipients and 7 liver recipients agreed to receive more information about the study and were contacted by telephone by a member of the research team providing further details (FB). Of these 74 patients, 38 accepted to participate and signed the informed consent (31 lung recipients and seven liver recipients). The heart transplant clinic referred five heart transplant recipients who had already consented to participate. From the group of 43 patients who had consented, 12 later declined to participate (9 lung recipients, 2 liver recipients and 1 heart recipient): 11 patients who had time constraints and one who wanted to participate in person, which was impossible during the COVID-19 pandemic. Participants received financial compensation for their time ($C30). Figure 1 summarises the recruitment process.

The timing of the two focus groups was determined by the participants’ and researchers’ availability. For the participants who could not be scheduled for a focus group, we conducted an individual interview. During the focus groups, participants were welcomed by the research assistant who had contacted them (FB). The focus groups were facilitated by the principal investigator (M-CF), who had experience in qualitative research and in conducting focus groups and was not involved in their transplant care. It was made clear from the beginning of the focus groups that whatever was addressed during the discussion would have no impact on their clinical care. The focus groups began with brief introductions of every participant, after which the research team made a short presentation on creative writing and medicine and explained the objectives of the study. Participants had an opportunity to ask questions about the presentation. Discussion then began and participants were invited to express their opinions and perspectives freely. The research assistant (FB) took notes during the focus groups. All the discussions were recorded and transcribed. One focus group lasted 60 min and the other one lasted 90 min.

An individual interview was proposed for anyone who could not attend a focus group. 15 interviews were conducted by telephone and five by videoconference by a member of the research team (FB, AA and M-FM). All the interviews were conducted in French. The interviews lasted around 30 min on average (ranging from 14 to 58 min) and were recorded and transcribed.

The issues covered during the interviews and focus groups were outlined in an interview guide with open-ended questions addressing the following topics: (1) the most significant moment in the interviewee’s transplantation journey; (2) experiences of sharing transplant self-narratives, (3) potential benefits or challenges of sharing self-narratives; (4) perspectives on publishing patients’ self-narratives and creative writings on a web platform and (5) sociodemographic data. Given that no study has been conducted of patients’ perspectives on sharing self-narratives pertaining to the transplantation journey, our interview guide was designed to elicit their experiences. The guide was developed by the research team from the literature on creative writing in medicine (M-CF, CM and SH). Consistent with qualitative methodology, the interview guide was modified during the study as new topics emerged from the interviews (see online supplemental material).^16^

10.1136/bmjopen-2024-096791.supp1 Supplementary data

#### Description of creative writing workshops

**Figure 2:**
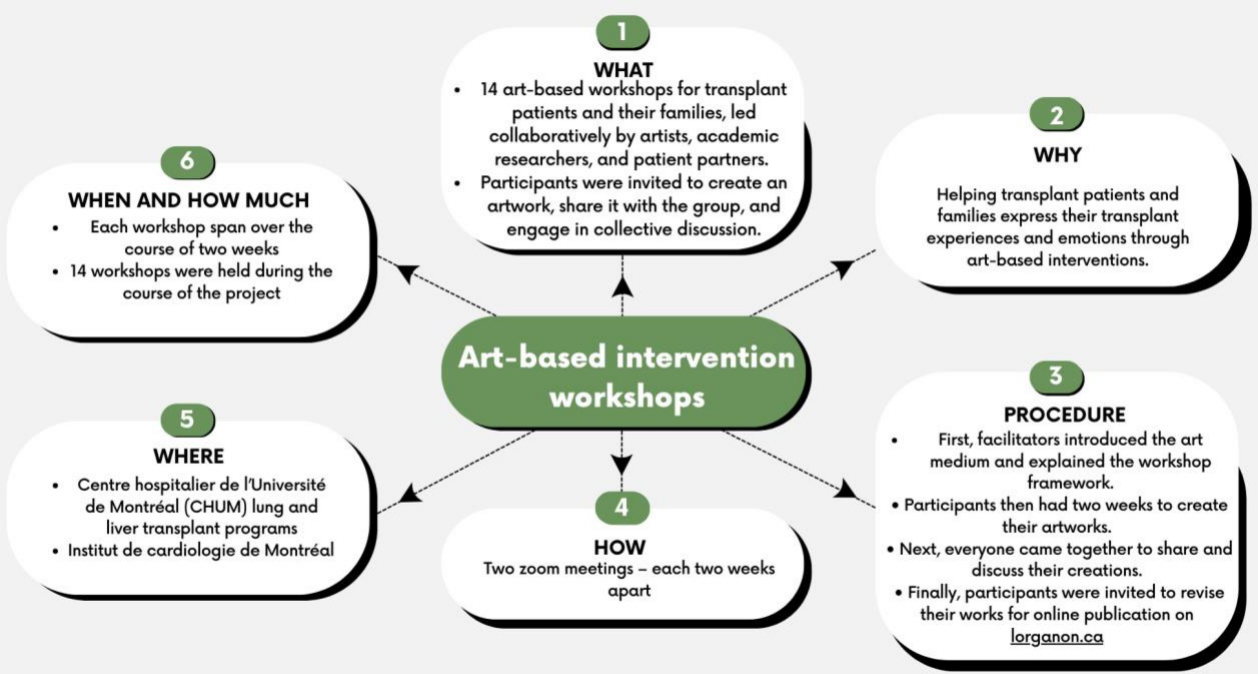
Description of the art-based intervention using the TIDieR checklist. TIDieR, Template for Intervention Description and Replication.

Between September 2020 and April 2022, we conducted fourteen 90-minute virtual creative writing workshops with heart, lung and liver transplant recipients. Each workshop was held 2 weeks apart and facilitated by two members of the research team with experience in workshop facilitation (SH and CM), along with one of fourteen guest artists. The artists represented a range of disciplines, including novel writing, poetry, comic art, puppetry and screenwriting, with the goal of exposing participants to diverse artistic media and experiences. Each artist participated in a single workshop (ie, two 90-minute sessions), while research team members were present throughout all sessions. Artists varied in their experience with ABIs, and most had not previously led ABIs with patients. Table 1 provides an overview of the titles (when applicable) and artistic disciplines of each intervention. Figure 2 presents a visual representation of the intervention, following the Template for Intervention Description and Replication (TIDieR) checklist.^18^

**Table 1:** Titles and art mediums of the workshops

Title of the art-based intervention	Art medium
No official title given	Literature
No official title given	Scriptwriting
No official title given	Literature
No official title given	Comics
Creating from Dreams: Transforming the Experience of the Body through Creative Writing	Literature
No official title given	Sound-texts
Memories	Text combined with other art forms (optional)
Memories and poems	Literature (poetry)
No official title given	Sound
L’importance du lieu dans l’écriture.	Literature
The world of zines	Zines
In My Body, There’s a Whole World…	Puppetry
Literary and Pictorial Self-Portraiture	Literature
Making a Scene: Writing Everyday Life	Literature

The first session, which was more theoretical, introduced the theme and artistic medium, allowing participants to familiarise themselves with the art form. Instructions for the creative activity were also provided. Participants then had 2 weeks to complete their creations. The second session served as a feedback forum, where participants could share their work, receive feedback, and discuss the meaning and message behind their creations, which often explored different aspects of their transplantation journey. Participants who consented were invited to publish their creative writing on the https://lorganon.ca website.

Although attendance was not restricted, participation averaged 8 to 10 individuals per workshop, fostering a small-group environment conducive to sharing and discussion. Transplant recipients were free to attend as many workshops as they wished. Each workshop invitation included information about the theme, artistic medium and guest artist, and was distributed to a list of interested individuals. As a result, attendance varied: some participants attended all sessions, others joined only one and some participated without actively sharing their artwork. No specific expectations were placed on participants, in keeping with the guiding principle that ABIs can engage both those who create art and those who experience it.^19^

#### Interviews conducted after the creative writing workshops

43 of the people who consented to participate (30 lung transplant recipients, 7 liver transplant recipients, 5 heart transplant recipients and 1 lung transplant candidate) agreed to receive an invitation to take part in the creative writing workshops, and 14 participated in at least one of the workshops (11 lung patients, 2 liver patients and 1 heart patient), which were held virtually between September 2020 and April 2022. Eight new transplant recipients (five lung recipients and three liver recipients) also joined the workshops (total of 22 participants: 16 lung recipients, 5 liver recipients and 1 heart recipient). All the participants (22) in the creative writing workshops were invited to be interviewed individually so that the team could gather their experiences. Nine lung transplant recipients, four liver transplant recipients and one heart transplant recipient agreed to participate and provided informed consent. Two patients (one heart recipient and one liver recipient) were lost to follow-up and six lung transplant recipients chose not to participate because of their health condition.

Interviews were conducted between October 2021 and April 2022. 17 interviews were conducted. 13 interviews were conducted by telephone and four by videoconference by a member of the research team (AA). Participants received financial compensation for their time ($C20). It was deemed more appropriate to conduct individual interviews because of the reduced number of post-workshop participants and for the sake of capturing their individual experience, rather than putting them back into the same context as the workshops, where they possibly had pre-existing dynamics. The interviews lasted between 10 and 30 min (about 16 min on average) and were recorded and transcribed. The following topics were addressed during the interviews: (1) experiences of participating in the creative writing workshops; (2) impact of the workshops and writing; (3) elements liked and disliked during the workshops; (4) experiences of sharing stories about their transplantation journey during the workshops; (5) how to post stories on a web platform and (6) sociodemographic data. The interview guide was developed by the research team (M-CF, CM and SH) (see online supplemental material)

#### Data analysis

We used a qualitative description approach to describe and analyse transplant recipients’ perspectives and experiences.^20^ The goal of this pragmatic approach was to use thematic analysis^21^ in order to remain as close as possible to the data collected during the interviews and provide a comprehensive summary of the topic studied.^22^ The latest version of the NVivo (Lumivero) software facilitated the analysis. Initial coding frames based on the interview grid were developed by M-FM, LD and AA. Codes were added and taken out based on the content of the interviews. The research team met frequently to discuss the coding frame and the data analysis. A member of the research team with expertise in qualitative methodology (M-FM) coded the interviews and no new codes were created after the eleventh interview for the preinterviews and focus groups before the creative workshops and after the eighth interview for the interviews conducted after the creative writing workshops.

During the coding process, a member of the research team who was new to qualitative work but is both a medical and literary student (LD), used the coding grid to code two interviews in order to assess inter-coder reliability mid-way through coding. LD then met with the team to discuss the importance and relevance of codes to the study until consensus was reached. Because MFM participated in 9 of the 14 ABI workshops, this step was considered essential to help maintain neutrality in the coding process. Afterward, MFM re-coded the previously coded material using the modified coding grid. The research team further engaged in collaborative discussions to resolve any discrepancies in theme identification and refinement, fostering a consensus-driven approach. The number of participants allowed for data saturation.^23^ At the end of the coding process, a team member (AA) with experience in qualitative methods, but who had not participated in the ABI workshops coded a portion of the raw data—purposively selecting transcripts from multiple stages of the coding process—to ensure coding accuracy and consistency across the dataset. The team then discussed the results until intercoder agreement was reached. None of the coders had personal experience as a transplant patient or as a caregiver for a transplant patient. However, both M-FM and AA have worked for several years on a qualitative research team studying the lived experiences of transplant candidates, patients and caregivers. Coded quotes were then organised by theme and subtheme.

#### Patient and public involvement

No patient partners were actively involved in this research project.

## Results

### Before the creative writing workshops: transplant patients’ perspectives on previous experiences of sharing self-narratives

#### Participants’ characteristics

11 lung transplant recipients took part in two virtual focus groups: seven in the first and four in the second. 11 lung transplant recipients, 5 liver transplant recipients and 4 heart transplant recipients took part in individual interviews before the creative writing workshops. Of these 31 participants, one lung transplant recipient did not complete the sociodemographic form. Among the remainder, 17 were female and 13 were male. They were in their 50s. The majority described themselves as White and 21 reported having a college or university level of education. Table 2 summarises participants’ characteristics.

**Table 2:** Characteristics of participants in preworkshop and postworkshop interviews

Characteristics	Preworkshop*	Postworkshop
	N=31 (%)	N=14 (%)
Type of organ transplant		
Lung transplant recipients	22 (71)	9 (64)
Liver transplant recipients	5 (16)	4 (29)
Heart transplant recipients	4 (13)	1 (7)
Sex		
Female/male/unknown	17(55)/13 (42)/1 (3)	8 (57)/6 (43)/(0)
Age (years)		
Mean±SD	54.9±12.9	55.1±15.9
	Range 29–72	Range 26–73
Race		
White	27 (87)	13 (93)
Other	2 (6)	0 (0)
Prefer not to answer	1 (3)	1 (7)
Unknown	1 (3)	0 (0)
Employment status		
Retired	10 (32)	6 (43)
Employed/self-employed	15 (48)	6 (43)
Unemployed	5 (16)	1 (7)
Unknown	1 (3)	1 (7)
Level of education		
College or university	21(68)	8 (57)
High school	8 (26)	3 (21)
Elementary school	1 (3)	2 (14)
Unknown	1 (3)	1 (7)
Annual family income		
More than US$100K	5 (16)	4 (29)
US$50K to US$99 999	13 (42)	3 (21)
Less than US$50K	7 (23)	5 (36)
Prefer not to answer	5 (16)	2 (14)
Unknown	1 (3)	0 (0)
Time since transplant		
(months±SD)	114.3±78.6	87.7±57.9
	Range 29–396	Range 9–188

*Only 30 participants completed the sociodemographic questionnaire.

#### Key moments during the transplantation journey and past experiences of peer support

Across various hospital settings, timings and types of organ, the transplant experiences reported by our participants share four common ‘key moments’: (1) being notified that they will need to undergo an organ transplant; (2) receiving the call once an organ is available; (3) the immediate post-transplant period and (4) becoming aware of one’s mortality. Table 3 presents interview excerpts.

**Table 3:** Key moments in the transplantation journey

Themes and interview excerpts	N=31
**Being notified that they will need to undergo an organ transplant** *“The most shocking moment, well, I think it was the moment when I was told that my liver was no longer good and that I was going to die, and that I was going to be on a waiting list for 6 months.”* (male liver recipient) *“It was when they told me that it was taking hold of me, that it was really taking hold of my heart. It’s gives you…it gives you a bit of a shock. In the end, when you’re faced with a fait accompli, the question is whether to let go and die or accept the heart.”* (male heart recipient)	**5**
**Receiving the call once an organ is available** *“…I got the phone call on the Monday, two days beforehand, saying I had to go in. You’re afraid, but at the same time you’re so happy, because for me it was just a matter of months then. She told me that it wasn’t a matter of years anymore, so we were really looking forward to it.”* (female liver recipient) *“But it’s really true that the call that came that night, well, I was called in the evening then I had to go into the hospital the next morning, but I think it was the evening, I would say it was the worst night of my life because you’re saying to yourself, well tomorrow it’s make or break, and you are hyper-aware of everything and the hours become very long. At the same time it goes by fast, but at the same time it feels long. You want to be positive and say to yourself, I’m going to come back, I’m going to come back in a few weeks, but at the same time you can’t ignore the fact that the risk is there and that these are major surgeries. So I think that that was one of the heaviest experiences in my life.”* (female lung recipient)	**5**
**The immediate post-transplant period** *“One moment I remember was when I was all alone in my hospital room and it had been maybe 2 or 3 days, I don’t know, a few days since my transplant, and then I was very, very vulnerable emotionally and then I thought about my donor and his family and it brought tears to my eyes. I was, it was mixed emotions because I was so happy, so grateful. I still am but I mean I was so like touched by the gesture, I was so like: Ah my god, it’s true, I’ve got my lungs now. There.”* (female lung recipient) *“Then after that, there was a kind of euphoria that set in once I’d been transplanted, woken up, everything was fine. In the weeks that followed, I remember I was laughing so hard I couldn’t stop, I was in a state of euphoria.”* (female lung recipient)	**5**
**Becoming aware of one’s mortality** *“There were times when I really thought I was going to die, and now I’m having recurring nightmares. It’s been three and a half years now, and it’s left a real mark on me, but it’s not so much a positive thing as a big change. I don’t know.”* (female lungs recipient) *“Oh my God!” (Hesitations) “There are a few. I think the most memorable moment was one morning when I woke up at home and I was having a pulmonary embolism and thought I was going to die.” (Sobbing*) (female lung recipient)	**6**

#### Previous experiences of sharing self-narratives

Most participants had experiences of sharing their stories prior to attending our creative workshops, in either spoken form (eg, in other groups or for an organisation) or written form (eg, a letter to their donor or a web platform). Participants reported that the content of their previous self-narratives focused on advice and encouragement, resulting in valorisation of experiential knowledge. Most participants felt that their previous experiences of sharing stories had been beneficial to their audience, namely by making others feel less isolated in their journey. However, two participants indicated that previous experiences of sharing self-narratives had been emotionally difficult, due to the act of opening up to others and being confronted by their immediate reactions.

Most participants also had previous experiences of hearing other transplant patients’ stories, in either spoken form (eg, in other support groups or in the hospital setting) or written form (eg, a web platform). Participants highlighted the following needs that were met through hearing others’ self-narratives: (1) access to experiential knowledge different from healthcare professionals’ expertise and (2) encouragement, hope and reassurance. However, it is worth noting that one participant reported feeling overwhelmed and ‘saturated’ after bearing witness to ‘too many narratives’. Table 4 presents interview excerpts.

**Table 4:** Experiences of sharing and hearing transplant self-narratives before the workshops

Interview excerpts	N=31
**Prior experience of sharing their own stories with other people** 1. Self-narratives are a source of advice and information for other patients *“People want to know how it’s gonna go, if you have any tips or tricks for them or if there’s anything they should know. They want to know about your experience, how things went for you.”* (female liver recipient) *“I encouraged several others to go through with the transplant. They would say, no, it’s too difficult, but I would tell them to take it one day at a time, they didn’t have a choice. It’s either a transplant or you die. You don’t have a choice. You’re better off accepting the transplant right off the bat.”* (female lung recipient)	7
2. Sharing transplant stories are a way to break the feeling of loneliness *“I think they feel less alone because even if you’re surrounded by family, sometimes family doesn’t truly understand what you’re going through.”* (female liver recipient)	12
3. Sharing transplant stories could have negative impact *“One person told me that I had scared them.”* (male lung recipient) *“I’d say that like the person I’d met face to face with her mother, well I saw that it had helped her a lot, but when I went back home, I had, it was like an emotional burden that was kind of heavy to bear. I’d found it a bit difficult there as it’s still quite draining, we can definitely say. I think it’s maybe not so for everyone, how can I put it? To open up so much here.”* (female lung recipient) *“I got angry because I felt that, at least for me, we’re all different, and then the person was waiting for a transplant, and then she was, I felt that she was kind of disrespectful toward the transplant request, and then I just stopped talking to her because it just made me angry and then it just made me mad to see that there was like no respect toward the donor afterwards when he had his transplant there.”* (female lung recipient)	3
**Prior experience of hearing other patients’ stories** 1. Access to experiential knowledge *“People want to know what’s going to happen. People want to know how it’s going to be, people want to know do you have stuff, is there anything I should know? They want to know about your experience, how it went for you too.”* (female liver recipient) *“She told me about her journey, her transplant. She too had a rather difficult transplant, and today she was well, she had a girlfriend and then her girlfriend had a child. So because there was that situation as well, there was a worry if I’m going to be immunosuppressed while living with a child who brings all the germs from daycare. So to have that reference and then she’d say: You just need to wash your hands more. It’s going to be all right then.”* (female lung recipient)	11
2. Encouragement, hope and reassurance *“It encouraged me… We all know that after a transplant, you’re a bit worried about the follow-up and what can happen, and when I saw that she was blossoming and that she had resumed her youthful life, because we were at the same stage of life, yes, it encouraged me.”* (female lung recipient) *“It was very encouraging to see how well she was doing. So that helped me a lot*. *…So I’d say what helped me get through it the most was to see… Well maybe to be aware or to meet people who have great stories there, great successes there. And when I say a great success, it’s important to be aware that there’s a lot of adapting to do afterwards, and then sometimes there are other little problems that arise, but the fact remains that people can resume their activities and continue to live as normal a life as possible, despite certain limitations.”* (female lung recipient) *“Well, all of their stories, they’ve all left their mark on me. Each person is different there. It was all positive, really more the hope that was there because there’s no hope when you’re waiting for lungs. I had a month to live, so I couldn’t wait for it to happen. It’s fun to see the positive results.”* (female lung recipient)	22

### Experiences of attending creative writing workshop

#### Participant characteristics

Nine lung transplant recipients, four liver transplant recipients and one heart transplant recipient took part in 17 individual interviews to document their experience of attending creative writing workshops. Three participants completed two interviews after attending different creative writing workshops. Eight of them were female and six were male. They were in their 50s and 13 were White. The majority had a post-secondary education level. The average time since the transplant was more than 7 years. Table 2 summarises the participants’ characteristics.

#### Positive and negative aspects of the creative writing workshops

Participants enjoyed the creative writing workshops. They reported that the creative writing workshops provided a respectful environment, with encouraging, open-minded and qualified facilitators. Many participants conveyed that the creative writing workshops allowed them to reflect and gain better insight on their transplantation journey. The group setting was also reported as a notable strength of the workshops, providing a space for peer support and a feeling of connection to others. Many participants also highlighted that the workshops were stimulating and inspired them to invest in their creative process. Importantly, several participants reported that their creative writings had a positive effect on them by enabling self-expression and, more specifically, emotional expression. Some participants also mentioned that the workshops strengthened their understanding and appreciation of organ donation. Of note, one participant who had undergone organ transplant several years prior to workshops emphasised that their primary interest in attending the workshops at the present time was to have a space for creativity that remains separate from their transplantation experience per se. Similarly, another participant indicated that they were attracted by the ‘opportunity to break free from the ‘sick talk’ and to instead delve into the arts’. Most participants would recommend the creative workshops to others who have undergone or are awaiting an organ transplant procedure.

The potential benefits of the creative writing workshops were clearly recognised by most participants. There was, however, a noteworthy heterogeneity regarding the participants’ experiences of the creative aspect of the workshops. While some participants reported feeling comfortable with the framework of the creative workshops, several of them reported feeling underqualified and intimidated by others’ artistic talents, with two participants describing a competitive environment among participants. Six participants expected the workshops to focus on the organ transplant experience specifically, and so they were disappointed by their perceived lack of testimonials through others’ artworks. In terms of logistics, participants commented on the length of the sessions and on the virtual setting. Two participants expressed that they would have liked more time for the group to share their creative writings, but one maintained that the sessions felt ‘lengthy’ enough as they were. Moreover, some participants commented on the barriers to fluid communication with other participants when using a virtual platform such as Zoom; for example, the lack of nonverbal cues, the lack of eye contact, and the technical difficulties. This may at least partly account for the five participants who had the impression that others took either too much or too little space during the workshops, which made them feel less comfortable than they would have liked. Table 5 presents interview excerpts.

**Table 5:** Positive and negative aspects of the creative writing workshops

Interview excerpts	N=17
**Positive aspects of creative writing workshops** *“There were some good discussions and even between the participants, it was very respectful and encouraging.”* (female lung recipient) *“It’s the type of workshop that allows you to share, to learn, to broaden your outlook and get different perspectives on what others have gone through vs what we have gone through ourselves, all while exploring different facets of art. Whether through writing or other media, being able to participate and chat with others and see what they think builds community.”* (female lung recipient) *“I liked how open and respectful the workshop atmosphere was. I also liked that, in a more general context, it was okay to participate if we wanted to, but also to hold back at times if it felt better. I liked the format: there was good discussion, everyone could participate, be heard, and circle back on what was created.”* (female lung recipient)	14
**Negative aspects of the creative writing workshops** *“I found it a bit strange that many participants said absolutely nothing and just looked at the zoom. Maybe it’s something I’m uncomfortable with because I don’t use zoom very often, so yes, it was strange for me to see other participants watching, but saying absolutely nothing.”* (female liver recipient) *“I don’t know why, when we were sharing our creations, I felt like it was such a personal thing. Maybe I’m getting it wrong and I don’t want to… but it seemed that others were trying to impress or that there was some kind of contest to see who was the sickest. I don’t know how to put it. I don’t want to speak badly about anyone, it was just the impression I got, that it was a contest to see who had it the hardest.”* (female lung recipient) *“I think it’s a bit of a shame that during the week when we went over everyone’s artwork, at times there were too many of us for everyone to share in the same room, so we had to split into two groups and then we didn’t get to see everyone’s results. It was a bit sad, because it’s interesting to see everyone’s artwork.”* (female liver recipient)	10

#### Therapeutic aspects of creative writing workshops and the associated peer support

The most frequently reported benefit of the creative process was its ‘therapeutic’ effect, especially through the meaning-making that results from conceiving and sharing their creative writing. Almost half of the participants reported being pleased to discover and experiment with new ways of creating during the workshops, which resulted for some in the exploration of a different perspective of their own selves. Several participants also stressed that the creative format of the workshops was crucial to the voicing of their self-narratives and associated emotions. The elaboration and subsequent sharing of their self-narratives made participants feel ‘proud’, ‘listened to’ and ‘valued as a human being’. Some participants also understood the creation of their artworks as a way to pay tribute and to value every stakeholder involved in the process of organ donation.

Participants who attended the creative writing workshops also reported ‘feeling connected to others’. Workshops were viewed as a way to break isolation and as a source of peer support. Several participants noticed that the positive experience of sharing their creative writings with other participants during the workshops empowered them to be vulnerable in a similar way with their spouse or family members. This feeling of belonging within a group or a community was the cornerstone of various other benefits, such as sharing of experiential knowledge, being touched by others’ narratives, putting into perspective and normalising the participants’ experiences, modelling resilience among participants, and cultivating open-mindedness and care.

While most of the participants’ feedback highlighted beneficial aspects of peer support, some participants acknowledged that to bear witness to testimonials that were at times ‘darker’ could be difficult, or even ‘triggering’. Two participants noted the importance of ‘detachment’ to avoid feeling overly distressed by others’ testimonials. Yet, one participant reported ‘detachment’ as an unwanted feeling which emerged on discerning self-pity in others’ testimonials. Table 6 presents interview excerpts.

**Table 6:** Impact of the creative writing workshops and peer support

Interview excerpts	n=31
**1.Therapeutic effect of the creative writing and sharing them** *“Sharing some pretty personal things allowed me to get rid of some feelings of guilt and it was the first time that I had spoken about certain subjects aloud, especially with people I didn’t know, and it helped me, because I didn’t feel judged.”* (female liver recipient) *“I think it helps you make sense of what you’ve gone through. It helps you put it into words, because not everyone has the same vocabulary or can express themselves easily. Sometimes, hearing others talk about something turns on a light bulb inside you and you’re like: Aha! That’s true, I’ve never thought about that or considered it quite like that before. So I think that even if it didn’t change anything, it helped me to make some sense of the different things that I’ve been through.”* (female lung recipient)	10
**2. Exploration of different perspectives of their own selves** *“I got an interesting writing experience out of it. I’m pretty shy, so writing allowed me to explore certain paths. At the time of the workshops, I was feeling emotional overload, so I closed up. All in all, I’m satisfied with the experience.”* (male lung recipient) *“I’ve spent my life in hospitals. I didn’t want my illness to define me, but I really started to feel that that’s what was happening. I’m more than my illness.”* (female lung recipient)	10
**3. Sharing creative writing is a source of pride** *“I think it helped me realize that every workshop helped me step outside of my comfort zone. All the creative writing, which is really not my forte, was a challenge, and that’s what I liked about it.”* (male lung recipient)	8
**4. Creative writing is a way to give back** *“It’s something that’s between us and also the fact that we have someone else’s organ inside us, we have a part of someone else within us and it’s also important to value this person who gave us life, it’s thanks to this person who donated their organ or to their family. We’re still here, we still exist, so it’s really important to pay tribute to them, yes, in a way.”* (female liver recipient) *“But I always have, not pleasure, but I like to talk about it and I’ve also done conferences for the Canadian Liver Foundation and then Partenaire Santé, and I find it so extraordinary that they managed to save my life and that I managed to have two transplants with two different livers in the space of 24 hours, that I’m always happy to be able to share that again because I think it’s a feat that they accomplished.”* (female liver recipient)	2
**4. Creative writing workshops let one feel connected and alleviate loneliness** *“Every story is important, and hearing different stories helps you feel less alone, because transplantees aren’t exactly common.”* (female liver recipient) *“I recognized my own story and it was an eye-opener. We think we’re unique, but after hearing those stories, we realize that we really aren’t so unique. So, when someone says something, I’ll say: Ah! It’s exactly the same for me. It doesn’t bother me not to be so unique, it’s actually comforting.”* (female lung recipient)	5
**6. Sharing narratives with other empower patients to be vulnerable with others** *“Well, I was very moved by what people were experiencing, and I was also moved by the perception they had of the two artworks I presented, and they understood the emotions I was conveying through them…… And then it was time to write the text, and I must admit that all the emotions came out at that point. …because it took me three times because my voice was shaking and then I was very, very moved to tell my story.”* (female liver recipient) *“It made me realize that I wasn’t the only one in much the same situation, and that we’d all been through difficult things for most of us, and that we’re all still here, still seeing our doctors, still having our treatments, but it’s like we’re going, we’re not giving up. It’s hard to see that there are all kinds of different situations, but they’re all difficult for everyone, depending on their circumstances. That’s it, I think it made me realize that a lot.”* (female liver recipient)	7
**7. Darker side of hearing other narratives** *“Sometimes it can be upsetting to meet others who, like us, are sick and have the same chronic illnesses and challenges. The whole mental health approach kind of surprises me. I’m quite shocked because there are some pretty depressed people in the group, even though it’s a different approach.”* (female liver recipient)	3

## Discussion

This is the first study to examine heart, lung and liver transplant recipients’ perspectives on the impact of creative writing workshops on their transplantation journey. We have previously reported the perspectives of kidney transplant candidates, kidney transplant recipients, and living donors on creative writing workshops.^15^ However, heart, liver and lung transplant recipients face different challenges than kidney patients do, since transplantation is a life-saving treatment for them—there is no other option, in the way that dialysis represents an option for patients with end-stage kidney disease. Heart, liver and lung transplant candidates have to face the possibility of their mortality if a transplant is not offered and also if there are serious adverse effects after transplantation. Becoming aware of one’s own mortality was a theme reported by participants in our current study, whereas it was not reported by the kidney transplant candidates and kidney transplant recipients in our earlier study.

As reported by kidney transplant patients, the call for a transplant was a key moment in their transplantation journey. In recent studies conducted with heart and lung transplant patients, the call for transplant was associated with fear and anxiety.^24 25^ The participants in our study also highlighted the immediate post-transplantation journey. Such experience aligns with the dominant narrative of transplantation, where the transplantation itself is a healing event. This is a linear and restorative narrative where transplantation will allow patients to return to health and have a second chance.^24 26^

For the participants in our study, sharing stories or hearing other patients’ stories during their transplantation journey wae described as valuable peer support and was viewed as a way to normalise their own experiences and put them into perspective. Also, sharing the same experiences of organ transplantation could make the listener more empathic and understanding than someone who has not gone through the transplant experience.^27^ Moreover, sharing stories and listening to other patients’ stories could help to better understand their identities as transplant patients but also as individuals. In this sense, peer support is a way of acquiring experiential knowledge.^28^ In a previous study, it was even stated that only someone who has gone through the process could understand what heart transplantation is.^29^ However, some patients did not want to be exposed to other patients’ stories because they believed that each patient’s experience is unique and so did not want to be scared by other patients’ bad experiences. This aligns with the results of other studies showing that peer support could be valuable for people awaiting a heart or heart/lung transplantation because peers could provide hope, first-hand information and reassurance. However, it was also reported that some patients did not want to hear about other patients’ stories because they did not want to be scared or anxious about the future.^25^

Previous studies have also examined the role of illness narratives and storytelling that create sense and coherence in the illness experience and serve as a tool for coping.^30 31^ Moreover, when patients share their stories with an audience, the act of storytelling lets them negotiate the meanings of the stories and redefine their identities.^27^ A recent study conducted with liver transplant recipients used the creation of short videos and writing exercises during workshops in order to develop a digital story.^32^ These workshops allow patients to expand their narrative possibilities and go beyond the archetypes associated with organ transplantation. Sharing the digital stories and commenting on other digital stories and videos allow participants in these workshops to create a sense of community and belonging.

For many participants, the creative writing workshops were described as ‘therapeutic’ and made them feel valued as holistic human beings. It was beyond the scope of this research project to assess the therapeutic aspect of creative writing workshops on the care trajectory of participants. However, this incidental reported effect could be explained partly by the peer support and partly by the fact that workshops allowed vulnerable patients to break their isolation during the anxiety-inducing period of the pandemics. That being said, the therapeutic quality of writing, for both mental and physical health, has been described previously by Pennebaker.^33^ He developed a technique of expressive writing that included the following principles: (1) the writer openly acknowledges and accepts their emotions; (2) they become able to express those emotions; (3) they construct a meaningful story and (4) writing provides a space for increased self-reflection.^34^ Given the standardised frame of expressive writing, it is distinct from creative writing where participants are encouraged to use different literary styles and use fiction and imagination in their writings. It is nonetheless striking to note the resemblance between the therapeutic elements described by our own participants and those reported by Pennebaker. A possible explanation is that expressive writing could be understood as a form of creative writing, hence resulting in similar cognitive and emotional processes being activated and similar therapeutic outcomes. A randomised controlled trial comparing expressive writing to neutral writing found peer support to be beneficial post-transplant only if there had been previous self-reflection and emotional expression.^35^ We may therefore hypothesise that emotional expression during our creative workshops was central to their potentially therapeutic yield.

Advances in the field of organ transplantation over the past decades have resulted in considerable improvement in post-transplant survival rates.^36^ However, organ transplantation is associated with existential challenges such as disruption in the sense of identity or selfhood. This disruption could be related to the alienation related to the disease leading to transplantation and the post-transplant care; to the symbolic nature of the transplanted organ, such as the heart; or to the bodily disruption and the feeling of interconnectedness with the person who donated the organs.^37 38^ Sharing narratives and peer support between transplant recipients was viewed by the participants as a way to normalise the transplantation journey and the changes of identity that could occur.

Finally, transplant programmes are increasingly seeking ways to support recipients in the post-transplant period.^39^ Research consistently shows that both physical and emotional health in transplant patients are closely linked to social support and physical activity, before and after transplantation.^40^ While many studies have highlighted the benefits of individual or group sports for transplant patients, physical activities may not suit every patient’s lifestyle or preferences.^41 42^ Transplant programmes could therefore explore alternative avenues for patient expression and social connection, such as ABI workshops. Like other group-based interventions, ABI workshops can foster (1) the sharing of knowledge and experiences within a community and (2) access to guidance and support.^43^ As such, they may contribute to long-term self-management among transplant patients.^44^ Moreover, these workshops are relatively low-cost and easy to implement, making them a promising option for new initiatives aimed at improving post-transplant quality of life and outcomes.

### Limitations

There are some limitations related to our study sample. All our participants came from two urban transplant centres in Quebec. These participants, both before and after the creative writing workshops, were mainly of White ethnicity with a college or university degree and a mean age of approximately 60 years. Because the creative writing workshops were conducted in French, we were not able to reach out to a greater diversity of patient ethnicities with a lower level of French literacy. The timing of the workshops, which were at night, could have been a barrier for some participants. The required time commitment may suit older, retired participants rather than young, working adults. The virtual format of the workshops, delivered over Zoom during the pandemic, also favoured patients with a stable or available Internet connection and digital literacy.

Another limitation in our data collection arises from the specificity of the multiple processes involved in the creative writing workshops. Although we were attentive to the reported experience of the creative writing process itself, most of the time the participants’ answers did not permit a clear distinction between the action of writing and that of sharing one’s writing and self-narratives in the workshop; the two actions were confounded together. Further studies are needed to tease out these different processes in relation to their reported therapeutic effects.

## Conclusions

In conclusion, our creative workshops provided a space dedicated to individual creative expression as well as the sharing of self-narratives and peer support. Our participants described the creative process as therapeutic, possibly as a result of increased insight, meaning-making, emotional expression and the feeling of being valued as a human being. Sharing of self-narratives and peer support were also found to be beneficial due to the feeling of connection with others, the sharing of experiential knowledge and the normalisation of experiences. Creative workshops that combine creative self-expression and peer support were found to be a feasible and beneficial intervention among transplant patients. More research is needed for the sake of studying the impacts of creative writing as an ABI, as opposed to other forms of storytelling: non-artistic (eg, digital storytelling, life review and reminiscence therapy)^45–47^ or psychotherapeutic (eg, expressive writing).^33 48–50^

## Supplementary Material

Reviewer comments

Author's manuscript

## Data Availability

Data are available on reasonable request. Data are available on reasonable request to the corresponding author.
