# Supplementary material for: Quebec lung, liver and heart transplant recipients’ perspectives on self-narratives and their experiences in creative writing workshops during the transplantation journey: a qualitative study
Source: BMJ Open. 2026 Jul 3;16(7):e096791. doi: 10.1136/bmjopen-2024-096791 (PMC13358346; doi:10.1136/bmjopen-2024-096791)
Supplement: Supplementary data [file bmjopen-16-7-s001.pdf]

Supplementary Material:

Pre-Workshop Focus group and Interview question Guide:

1. What is the most significant moment in your transplant story?
2. Have you ever shared your story with other patients?
   a. If yes:
   i. What did you share? In what context?
   ii. What impact did this story have on the other person? On you?
   b. If not, why?
3. What is your experience of receiving stories from transplanted patients?
   a. At what point did you receive these stories?
   b. What was the impact of these stories on your journey?
   c. What did you appreciate about these stories?
4. In your opinion, can patient stories be beneficial?
   a. How?
   b. What elements should be included in these stories?
   c. At what point in the care trajectory?
   i. During pre-transplant evaluation?
   ii. During registration and waiting period?
   iii. During the transplant and immediate post-op?
   iv. The first year post-transplant?
   v. Throughout the transplant journey?
   vi. During graft loss?
   d. Who should be the narrators of these stories?
5. Do you recall transplant stories told through media such as movies, novels, comics, or clips?
   a. What reactions did you have when seeing, reading, or hearing these stories?
6. What do you think about the idea of developing a web platform that showcases the diversity of transplant patient journeys?
7. If we were to develop a web platform with patient stories, what form should these stories take?
8. What do you think about healthcare providers stories? Would they be useful? What should they include?
9. How can patient stories help other patients cope with transplantation?

Post-Workshop Interview Question Guide

1. Which creative writing workshops did you participate in?
2. What has been your experience with the creative writing workshops?
3. Which workshop did you enjoy the most? Why?
4. What did you gain from these workshops?
5. What aspects of these workshops did you like?
6. What aspects did you like less and think could be improved?
7. What has been your experience of sharing your transplant story with others?
8. What impact did the stories of other participants have on you?
9. How do you envision your texts and creations on the web platform?
10. How have these workshops affected you?
11. How have these workshops changed your experience of transplantation or illness?
12. Would you recommend such workshops to someone close to you?
13. Do you have any other comments you would like to share with me?
