# Supplementary material for: Quebec lung, liver and heart transplant recipients’ perspectives on self-narratives and their experiences in creative writing workshops during the transplantation journey: a qualitative study
Source: BMJ Open. 2026 Jul 3;16(7):e096791. doi: 10.1136/bmjopen-2024-096791 (PMC13358346; doi:10.1136/bmjopen-2024-096791)
Supplement: Reviewer comments [file bmjopen-2024-096791.reviewer_comments.pdf]

PEER REVIEW HISTORY

BMJ Open publishes all reviews undertaken for accepted manuscripts. Reviewers are asked to complete a checklist review form and are provided with free text boxes to elaborate on their assessment. These free text comments are reproduced below.

ARTICLE DETAILS

Title (Provisional)

Quebec Lung, Liver, and Heart Transplant Recipients’ Perspectives on Self-Narratives and Their Experiences in Creative Writing Workshops During the Transplantation Journey: A Qualitative Study

Authors

Ducharme, Laurence; Malo, Marie-Françoise; Affdal, Aliya; Ballesteros, Fabian; Laneuville, Laurence; Brassard, Leonore; Gagnon-Chainey, Benjamin; Millot, Pascale; Mavrikakis, Catherine; Harel, Simon; Fortin, Marie-Chantal

---

VERSION 1 - REVIEW

**Reviewer**	**1**
**Name**	**Gibbons, Andrea**
**Affiliation**	**University of Winchester Department of Psychology**
**Date**	**16-Dec-2024**
**COI**	**None**

This paper focuses on the benefits of creative writing for those in receipt of a solid organ transplant.

It provides a unique perspective on how those with a liver/lung/heart transplant make sense of their experiences, in the context of creative writing. I think the study is important, but there are some details that would improve this paper.

I think the introduction could be improved; for example why is specific info on the workshops detailed in the introduction? A little more detail on the rationale for the study, or a more general overview of the research / research questions would be more useful.

The method could be more clearly structured. I think it needs reworking so that it is clear what happens and when. How many of the 43 people who consented to take part, actually took part in the interviews /focus groups AND the workshops and interviews?

Why were 14 professional writers used, and not fewer (with them running more than one workshop)? What was the content of the workshops? The quotations talk about artwork and writing; more information about what participants were asked to do would be useful.

Why were 3 participants allowed to give 2 interviews each? Why did they attend different workshops?

The analysis feels a bit too descriptive for a qualitative study. For example, the use of data saturation and coder agreement is increasingly being seen as inappropriate for various qualitative methods, especially for thematic analysis. A way to address this would be to be much clearer on your own positioning (e.g. ontology/epistemology) and give some more detail on the specific steps of your analysis. That way it will be clearer that this is more of a content/descriptive analysis from a more realist perspective. I appreciate that you have included some detail (and are not trying to present it as anything other than this), but my concern is that those not familiar with qualitative methods will presume from your paper that this is how thematic analysis should be conducted.

Reviewer **2**

Name **Nubret, Karine**

Affiliation **University Hospital Centre Bordeaux, Heart failure department, heart transplantation and MCS unit**

Date **03-Aug-2025**

COI **None**

This qualitative research study aims to explore the role of patients’ self-narratives and creative writing workshops during their transplantation journey. The data collection—comprised of patients’ words and observations—is appropriate and aligned with the methodology of qualitative research.

However, the study’s objectives are not clearly defined. The potential therapeutic effects of the intervention deserve to be further elaborated, particularly regarding any pre- and post-workshop evaluation of patients’ perceptions of their personal narratives and their care trajectory.

The impact of the workshop itself should be described in greater detail. Moreover, the relevance and potential value of such an intervention for the broader medical and scientific community are insufficiently highlighted to justify further exploration or replication in other contexts.

The authors appropriately address the triangulation of perspectives and peer validation. However, there is no mention of how reflexivity was incorporated into the research process, which is a key component in qualitative inquiry.

These aspects require further clarification and development before the manuscript can be considered for publication.

VERSION 1 - AUTHOR RESPONSE

REVIEWER 1 COMMENTS TO THE AUTHOR:

1. It provides a unique perspective on how those with a liver/lung/heart transplant make sense of their experiences, in the context of creative writing. I think the study is important, but there are some details that would improve this paper.

We thank the reviewer for their thoughtful comment on our manuscript.

2. I think the introduction could be improved; for example why is specific info on the workshops detailed in the introduction? A little more detail on the rationale for the study, or a more general overview of the research / research questions would be more useful.

Thank you for this insightful comment. In the revised version, we have expanded the Introduction to provide a clearer rationale for the study and a more comprehensive overview of the research objectives and questions. We also clarified the role of the workshops within the broader study, ensuring that their mention in the Introduction serves primarily to contextualize the research rather than to describe procedural details. (See pp. 5-6)

In parallel, the Methods section has been enhanced to include more detailed information about the workshops themselves, including their structure and content. (See pp. 9-10)

3. The method could be more clearly structured. I think it needs reworking so that it is clear what happens and when. How many of the 43 people who consented to take part, actually took part in the interviews /focus groups AND the workshops and interviews?

Please see our response to the editor's comment 7. We have also added Figure 1 to summarize the recruitment process.

4. Why were 14 professional writers used, and not fewer (with them running more than one workshop)? What was the content of the workshops? The quotations talk about artwork and writing; more information about what participants were asked to do would be useful.

Thank you for this thoughtful and constructive comment. We have added information in the Methods section about the artists and writers who facilitated the workshops. The decision to invite 14 different professional artists was made to offer a broad range of artistic methods and styles, thereby attracting participants with diverse interests and creative affinities. Each artist was invited to lead a single two-session workshop to ensure variety across workshops and to expose participants to multiple forms of artistic expression. However, the research team attended all workshops, ensuring continuity across sessions and consistency in data collection.

We appreciate the reviewer's point regarding the potential benefit of having some artists facilitate multiple workshops. This is an excellent suggestion that will be considered in the design of future art-based interventions (ABIs), as it may provide valuable continuity and allow deeper engagement between artists and participants.

Additional information has also been added about what participants were asked to do during the workshops. A new Table 1 has been included, listing the titles (when available) of the workshops and the type of art explored in each. We recognize that it would have been valuable

to include the specific creative prompts or instructions used in each workshop; however, as these were communicated orally rather than in written form, it was not possible to retrieve and systematically include them in the table.

5. Why were 3 participants allowed to give 2 interviews each? Why did they attend different workshops?

Thank you for this question. Participants were allowed to attend multiple workshops, and three participants were invited to take part in two post-ABI interviews. This approach aimed to explore whether exposure to different art forms might elicit distinct reflections or emotional responses, or whether participating at multiple time points might influence their experience or its perceived impact. However, since no meaningful differences were observed based on art type or repeated participation, these findings were not included in the present article.

6. *The analysis feels a bit too descriptive for a qualitative study. For example, the use of data saturation and coder agreement is increasingly being seen as inappropriate for various qualitative methods, especially for thematic analysis. A way to address this would be to be much clearer on your own positioning (e.g. ontology/epistemology) and give some more detail on the specific steps of your analysis. That way it will be clearer that this is more of a content/descriptive analysis from a more realist perspective. I appreciate that you have included some detail (and are not trying to present it as anything other than this), but my concern is that those not familiar with qualitative methods will presume from your paper that this is how thematic analysis should be conducted.*

Thank you for this very helpful and constructive comment. We have revised the Data Analysis subsection of the Methods to enhance clarity regarding our analytical stance and to include a description of the positionality of the three primary coders involved in the analysis. This addition more explicitly situates the analysis within its epistemological and interpretive context, helping readers better understand the lens through which the data were examined.

We have also expanded the description of the coding process by providing a more detailed overview of the specific steps followed during data analysis. This revision aims to increase transparency, clarify the rationale behind our methodological choices, and acknowledge potential sources of bias or interpretive influence inherent in the analytic process. We hope these additions offer greater methodological clarity and align more closely with current standards in qualitative research. (See pp.12-13)

REVIEWER 2 COMMENTS TO THE AUTHOR :

1. However, the study's objectives are not clearly defined. The potential therapeutic effects of the intervention deserve to be further elaborated, particularly regarding any pre- and post-workshop evaluation of patients' perceptions of their personal narratives and their care trajectory.

Thank you for this thoughtful comment. In the revised manuscript, we have clarified that the therapeutic effects observed were incidental outcomes of the workshops, rather than the primary focus of the study. Evaluating or measuring therapeutic impact was beyond the scope of this research. This point has been explicitly addressed in the Discussion section. (See p. 20)

2. The impact of the workshop itself should be described in greater detail. Moreover, the relevance and potential value of such an intervention for the broader medical and scientific community are insufficiently highlighted to justify further exploration or replication in other contexts.

Thank you for this valuable comment. In response, we have expanded the Discussion section to include a more detailed reflection on the impact of the workshops on participants, drawing on both observations and qualitative findings from the study. We have also added a concluding paragraph that highlights the potential applications and relevance of ABI workshops within transplant programs, as well as their possible value for the broader medical and scientific community. This addition underscores how such interventions can complement existing psychosocial and patient engagement strategies.(See p. 22)

3. *The authors appropriately address the triangulation of perspectives and peer validation. However, there is no mention of how reflexivity was incorporated into the research process, which is a key component in qualitative inquiry.*

Thank you for this helpful comment. We have revised the Data Analysis section to clarify our analytical positioning and the positionality of the three primary coders. We also added a more detailed description of the coding steps to enhance transparency and to highlight potential biases and interpretive perspectives embedded in the analysis. (See pp. 12-13)

1. Hoffmann TC, Glasziou PP, Boutron I, Milne R, Perera R, Moher D, et al. Better reporting of interventions: template for intervention description and replication (TIDieR) checklist and guide. Bmj. 2014;348:g1687.

VERSION 2 - REVIEW

**Reviewer**	**1**
**Name**	**Gibbons, Andrea**
**Affiliation**	**University of Winchester Department of Psychology**
**Date**	**17-Nov-2025**
**COI**	

The authors have adequately addressed my comments.
