# Supplementary material for: Quebec lung, liver and heart transplant recipients’ perspectives on self-narratives and their experiences in creative writing workshops during the transplantation journey: a qualitative study
Source: BMJ Open. 2026 Jul 3;16(7):e096791. doi: 10.1136/bmjopen-2024-096791 (PMC13358346; doi:10.1136/bmjopen-2024-096791)
Supplement: Author's manuscript [file bmjopen-2024-096791.draft_revisions.pdf]

BMJ Open

BMJ Open is committed to open peer review. As part of this commitment we make the peer review history of every article we publish publicly available.

When an article is published we post the peer reviewers' comments and the authors' responses online. We also post the versions of the paper that were used during peer review. These are the versions that the peer review comments apply to.

The versions of the paper that follow are the versions that were submitted during the peer review process. They are not the versions of record or the final published versions. They should not be cited or distributed as the published version of this manuscript.

BMJ Open is an open access journal and the full, final, typeset and author-corrected version of record of the manuscript is available on our site with no access controls, subscription charges or pay-per-view fees (http://bmjopen.bmj.com).

If you have any questions on BMJ Open's open peer review process please email
info.bmjopen@bmj.com

BMJ Open

Lung, Liver, and Heart Transplant Recipients' Perspectives on the Role of Self-Narratives and Creative Writing Workshops: A Qualitative Study

Journal:	*BMJ Open*
Manuscript ID	bmjopen-2024-096791
Article Type:	Original research
Date Submitted by the Author:	18-Nov-2024
Complete List of Authors:	Ducharme, Laurence ; Université de Montréal Faculté de Médecine<br>Malo, Marie-Françoise; Université de Montréal, École de santé publique, Bioethics Department<br>Affdal, Aliya; Université de Montréal, École de santé publique, Bioethics Department<br>Ballesteros, Fabian; Centre Hospitalier de l'Université de Montréal<br>Laneuville, Laurence; Université de Montréal Faculté de Médecine<br>Brassard, Leonore; Université de Montréal, Chaire McConnell-Université de Montréal sur les récits de don et de la vie en contexte de soins<br>Gagnon-Chainey, Benjamin; Université de Montréal Faculté des Arts et des Sciences<br>Millot, Pascale; Université de Montréal Faculté des Arts et des Sciences<br>Mavrikakis, Catherine; Université de Montréal Faculté des Arts et des Sciences<br>Harel, Simon; Université de Montréal Faculté des Arts et des Sciences<br>Fortin, Marie-Chantal; Centre de Recherche du CHUM,
Keywords:	TRANSPLANT MEDICINE, QUALITATIVE RESEARCH, Peer Group, Literature

SCHOLARONE™
Manuscripts

I, the Submitting Author has the right to grant and does grant on behalf of all authors of the Work (as defined in the below author licence), an exclusive licence and/or a non-exclusive licence for contributions from authors who are: i) UK Crown employees; ii) where BMJ has agreed a CC-BY licence shall apply, and/or iii) in accordance with the terms applicable for US Federal Government officers or employees acting as part of their official duties; on a worldwide, perpetual, irrevocable, royalty-free basis to BMJ Publishing Group Ltd ("BMJ") its licensees and where the relevant Journal is co-owned by BMJ to the co-owners of the Journal, to publish the Work in this journal and any other BMJ products and to exploit all rights, as set out in our licence.

The Submitting Author accepts and understands that any supply made under these terms is made by BMJ to the Submitting Author unless you are acting as an employee on behalf of your employer or a postgraduate student of an affiliated institution which is paying any applicable article publishing charge ("APC") for Open Access articles. Where the Submitting Author wishes to make the Work available on an Open Access basis (and intends to pay the relevant APC), the terms of reuse of such Open Access shall be governed by a Creative Commons licence – details of these licences and which Creative Commons licence will apply to this Work are set out in our licence referred to above.

Other than as permitted in any relevant BMJ Author's Self Archiving Policies, I confirm this Work has not been accepted for publication elsewhere, is not being considered for publication elsewhere and does not duplicate material already published. I confirm all authors consent to publication of this Work and authorise the granting of this licence.

For peer review only

TITLE: Lung, Liver, and Heart Transplant Recipients' Perspectives on the Role of Self-Narratives and Creative Writing Workshops: A Qualitative Study

AUTHORS: Laurence Ducharme, MD[1], Marie-Françoise Malo, PhD(c)[2,] Aliya Affdal, MSc[2,] , Fabian Ballesteros, MSc[3], Laurence Laneuville, MD, MA[4],Léonore Brassard, PhD[5], Benjamin Gagnon-Chainey, PhD[5], Pascale Millot, PhD(c)[5,6], Catherine Mavrikakis, PhD[5,6], Simon Harel, PhD[5,7], Marie-Chantal Fortin, MD, PhD [1,2,3,5,8]

1. Faculty of Medicine, Université de Montréal, Montréal, Canada
2. Bioethics Program, École de santé publique de l'Université de Montréal, Montréal, Canada
3. Centre de recherche du CHUM, Montréal, Canada
4. Faculty of Medicine, Département de psychiatrie et d'addictologie, Université de Montréal, Montréal, Canada
5. Chaire McConnell-Université de Montréal sur les récits de don et de la vie en contexte de soins, Université de Montréal, Montréal, Canada
6. Département des littératures de langues françaises, Faculté des arts et sciences, Université de Montréal, Montréal, Canada
7. Département de littératures et de langues du monde, Faculté des arts et sciences, Université de Montréal, Montréal, Canada
8. Canadian Donation and Transplantation Research Program, Edmonton, Canada

CORRESPONDING AUTHOR:
Marie-Chantal Fortin, MD, PhD, FRCPC
Centre de recherche du CHUM
900, rue St-Denis, Salle R12-418
Montréal (Québec) H2X 0A9
514-890-8000 ext. 25712
marie-chantal.fortin.med@ssss.gouv.qc.ca

WORD COUNT : 4,500 WORDS

For peer review only

ABSTRACT

Background: Although solid organ transplantation is a life-saving procedure, it is also associated with numerous challenges for patients. Creative writing has been described as a therapeutic tool for patients with chronic disease. Between 2020 and 2022, we held creative writing workshops with patients and professional writers. During these workshops, patients were invited to explore different aspects of their transplantation journey through different literary styles.

Objectives: The aim of this study is to understand lung, liver and heart transplant patients' perspectives on the role of patient self-narratives and creative writing workshops during their transplantation journey, and collect testimonies on their experiences in the workshops.

Design: Focus groups and individual interviews.

Setting: The Centre hospitalier de l'Université de Montréal (CHUM) lung and liver transplant programs and the Montreal Heart Institute (MHI) heart transplant program.

Participants: Lung, liver and heart transplant recipients attending the CHUM and the MHI transplant clinic between September 2020 and August 2022.

Methods: Before the creative writing workshop, we conducted two focus groups and individual interviews with 22 lung recipients, five liver transplant recipients and four heart transplant patients. After their participation in the creative workshop, we conducted 17 semi-structured interviews with nine lung transplant recipients, four liver transplant recipients and one heart transplant recipient (three participants participated twice in interviews). Interviews were transcribed for subsequent qualitative description analyses.

Results: Most participants had previous experiences of sharing their transplant stories and considered that a web-based platform where transplant patients share their stories could be highly beneficial for future transplant patients and the public. The creative workshops were described as "therapeutic" since they let participants express their emotions and realities. Peer support and connection to other people

For peer review only - http://bmjopen.bmj.com/site/about/guidelines.xhtml

contributed to normalize their transplant journey and helped participants put their experiences into perspective.

Conclusion: Creative writing workshops and peer support through the sharing of self-narratives are a feasible approach that participants have described as beneficial. Future studies are needed to assess the benefits of patients' creative writings on other patients and on caregivers.

For peer review only

STRENGTHS AND LIMITATIONS:

- First study to examine heart, lung and liver transplant recipients' perspectives on the impact of creative writing workshops on their transplantation journey.
- One limitation of our study is that participants were mainly White and highly educated.

FUNDING

This research project was funded by the Chaire McConnell-Université de Montréal sur les récits de don et de la vie en contexte de soins and by the Audace Program of the Fonds de recherche Québec. Dr. Marie-Chantal Fortin is a research scholar for the Fonds de recherche du Québec – Santé (FRQS) and an investigator for the Canadian Donation and Transplantation Research Program.

COMPETING INTERESTS

Laurence Ducharme, Marie-Françoise Malo, Laurence Laneuville, Fabian Ballesteros, Alyia Affdal, Léonore Brassard, Benjamin Gagnon-Chainey, Pascale Millot, Catherine Mavrikakis, Simon Harel and Marie-Chantal Fortin have no competing interests to declare.

For peer review only

INTRODUCTION

Heart, lung and liver transplantations are treatments of choice for end-stage cardiac, lung or liver disease.(Beilby, Moss-Morris, and Painter 2003) Living with a life-threatening illness leads to personal and social changes that can be difficult to cope with.(Cierpka, Małek, and Horodeńska 2015, Anderson et al. 2017) Despite reporting a better quality of life after transplantation,(Duffy et al. 2010) emotional and psychological impacts of a transplant can remain long after the physical improvement of the recipient.(Dew and DiMartini 2005, Rosenberger et al. 2016)

Storytelling and art-based intervention have gained a lot of traction in recent years as ways to bolster patients' recovery journey.(Bruce, Daudt, and Breiddal 2018) These approaches have been shown to benefit patients with mental illnesses,(Romm, Synnes, and Bondevik 2022, Synnes, Romm, and Bondevik 2021) cancer patients,(Forzoni et al. 2010) and patients with chronic illnesses.(Kelly and Dickinson 1997, Currier and Zimmerman 2019) Art-based interventions can encompass several different art forms—from creative writing, to visual arts, to music.(Romm, Synnes, and Bondevik 2022) Storytelling can help patients make sense of their emotions and can offer a setting and a framework for them to explore difficult aspect of their illness journey.(Marini 2016) The subsequent dissemination of these stories may also help others during their own journey of care towards and after transplantation.(Ho et al. 2022) Our research group has already demonstrated, in previous work, the benefits of creative writing workshops and the diffusion of these self-narratives and creative writings for kidney transplant recipients, kidney transplant candidates and living kidney donors.(Laneuville et al. 2022) However, there is no data on the perspectives of heart, lung and liver transplant recipients on the role of creative writings and their experiences of creative writing workshops.

For peer review only - http://bmjopen.bmj.com/site/about/guidelines.xhtml

Between September 2020 and April 2022, we conducted fourteen 90-minute virtual creative writing workshops with heart, lung and liver transplant recipients. Each workshop was held two weeks apart by two members of the research team (SH and CM) and by one of 14 different professional writers (their disciplines included novelist, poet, comic artist, puppeteer and screenwriter). During the first session, one member of the research team and the invited professional writer introduced the theme and a short text to illustrate it. They also gave indications for the type of creative writing proposed. In the second session, transplant recipients were invited to share their work based on the indications provided during the first session, which could explore different aspects of their transplantation journey. Participants who agreed were invited to post their creative writing on a website.

The objective of this study was two-fold. First, before the creative writing workshops took place, the patients were invited to discuss their perspectives on the experience of sharing self-narratives related to their transplantation journey. Second, after their participation in workshops, we recorded the patients' experiences of attending the workshops which included sharing their self-narratives and creative writings.

For peer review only

For peer review only - http://bmjopen.bmj.com/site/about/guidelines.xhtml

METHODS

This study was exploratory in nature and used semi-structured interviews and focus groups with heart, lung and liver transplant recipients. It employed the COREQ checklist for reporting qualitative research.(Tong, Sainsbury, and Craig 2007) (See the detailed checklist) We conducted individual interviews and focus groups before participants attended the creative writing workshops, and individual interviews after the workshops. The CHUM institutional research ethics committee (CHUM CE20.206) approved the study; and all the participants gave informed consent to take part in the pre-workshop focus groups and in the interviews held before and after the workshops. After the workshops, there was a separate consent process regarding the decision to post the creative writings on the website anonymously or not.

Interviews and focus groups conducted before the creative writing workshops

Recruitment for focus groups and interviews was carried out between December 2020 and August 2022. Convenience sampling was the chosen approach for recruiting participants. French-speaking adults who were recipients of a heart, lung or liver transplant, and were followed at the two transplant clinics, were invited to participate by means of letters, posters, emails and flyers distributed at the transplant clinic. Directors of the lung and liver transplant programs sent an invitation email to 377 patients (358 lung recipients and 19 liver recipients), followed by a telephone call made by a member of the research team (FB or MFM). The heart transplant clinic approached the patients directly and obtained their informed consent before referring them to the research coordinator (FB).

Sixty-seven lung recipients and seven liver recipients agreed to receive more information about the study and were contacted by telephone by a member of the research team providing further details (FB). Of these 74 patients, 38 accepted to participate and signed the informed consent (31 lung recipients and seven liver recipients). The heart transplant clinic referred five heart transplant recipients who had

For peer review only - http://bmjopen.bmj.com/site/about/guidelines.xhtml

already consented to participate. From the group of 43 patients who had consented, 12 later declined to participate (nine lung recipients, two liver recipients and one heart recipient): 11 patients who had time constraints, and one who wanted to participate in person, which was impossible during the COVID pandemic. Thus, a total of 31 patients participated in the study: 22 lung transplant recipients, five liver transplant recipients, and four heart transplant recipients. Participants received financial compensation for their time (CAN$30). Figure 1 summarizes the recruitment process.

Eleven participants, all lung transplant recipients, took part in two virtual focus groups: seven in the first and four in the second. The timing of the focus groups was determined by the participants' and researchers' availability. For the participants who could not be scheduled for a focus group, we conducted an individual interview. During the focus groups, participants were welcomed by the research assistant who had contacted them (FB). The focus groups were facilitated by the principal investigator (MCF), who had experience in qualitative research and in conducting focus groups and was not involved in their transplant care. It was made clear from the beginning of the focus groups that whatever was addressed during the discussion would have no impact on their clinical care. The focus groups began with brief introductions of every participant, after which the research team made a short presentation on creative writing and medicine and explained the objectives of the study. Participants had an opportunity to ask questions about the presentation. Discussion then began and participants were invited to express their opinions and perspectives freely. The research assistant (FB), took notes during the focus groups. All the discussions were recorded and transcribed. One focus group lasted 60 minutes and the other one lasted 90 minutes.

An individual interview was proposed for anyone who could not attend a focus group. Twenty participants were interviewed individually: 11 lung transplant recipients, five liver transplant recipients, and four heart transplant recipients. Fifteen interviews were conducted by telephone and five by

For peer review only - http://bmjopen.bmj.com/site/about/guidelines.xhtml

videoconference by a member of the research team (FB, AA and MFM). All the interviews were conducted in French. The interviews lasted around 30 minutes on average (ranging from 14 to 58 minutes) and were recorded and transcribed.

The issues covered during the interviews and focus groups were outlined in an interview guide with open-ended questions addressing the following topics: i) the most significant moment in the interviewee's transplantation journey; ii) experiences of sharing transplant self-narratives; iii) potential benefits or challenges of sharing self-narratives; iv) perspectives on publishing patients' self-narratives and creative writings on a web platform; and v) socio-demographic data. Given that no study has been conducted of patients' perspectives on sharing self-narratives pertaining to the transplantation journey, our interview guide was designed to elicit their experiences. The guide was developed by the research team from the literature on creative writing in medicine (MCF, CM, SH). Consistent with qualitative methodology, the interview guide was modified during the study as new topics emerged from the interviews.(Tong, Sainsbury, and Craig 2007)

Interviews conducted after the creative writing workshops

Forty-three of the people who consented to participate (30 lung transplant recipients, seven liver transplant recipients, five heart transplant recipient and one lung transplant candidate) agreed to receive an invitation to take part in the creative writing workshops, and 14 participated in at least one of the workshops (11 lung patients, two liver patients and one heart patient), which were held virtually between September 2020 and April 2022. Eight new patients (five lung recipients and three liver recipients) also joined the workshops (total of 22 participants: 16 lung recipients, five liver recipients and one heart recipient). All the participants (22) in the creative writing workshops were invited to be interviewed individually so that the team could gather their experiences. Nine lung transplant recipients, four liver transplant recipients and one heart transplant recipient agreed to participate and provided informed

For peer review only - http://bmjopen.bmj.com/site/about/guidelines.xhtml

consent. Two patients (one heart recipient and one liver recipient) were lost to follow-up and six lung transplant recipients chose not to participate because of their health condition.

Interviews were conducted between October 2021 and April 2022. Seventeen interviews were conducted with 14 participants (nine lung transplant recipients, four liver transplant recipients, one heart transplant recipient). Three participants gave two interviews after different creative writing workshops. Thirteen interviews were conducted by telephone and four by videoconference by a member of the research team (AA). Participants received financial compensation for their time (CAN$20). It was deemed more appropriate to conduct individual interviews because of the reduced number of post-workshop participants, and for the sake of capturing their individual experience, rather than put them back into the same context as the workshops, where they possibly had pre-existing dynamics. The interviews lasted between 10 and 30 minutes (about 16 minutes on average) and were recorded and transcribed. The following topics were addressed during the interviews: i) experiences of participating in the creative writing workshops; ii) impact of the workshops and writing; iii) elements liked and disliked during the workshops; iii) experiences of sharing stories about their transplantation journey during the workshops; iv) how to post stories on a web platform; v) sociodemographic data. The interview guide was developed by the research team (MCF, CM, SH).

Data analysis

We used a qualitative description approach to describe and analyze transplant recipients' perspectives and experiences.(Sandelowski 2010) The goal of this pragmatic approach was to use thematic analysis(Green and Thorogood 2014) in order to remain as close as possible to the data collected during the interviews and provide a comprehensive summary of the topic studied.(Sandelowski 2000) The latest version of the NVivo (Lumivero) software facilitated the analysis. An initial coding frame based on the interview grid was developed. Codes were added and taken out based on the content of the interviews.

For peer review only - http://bmjopen.bmj.com/site/about/guidelines.xhtml

The research team met frequently to discuss the coding frame and the data analysis. A member of the research team with expertise in qualitative methodology (MFM) coded the interviews and no new codes were created after the eleventh interview for the pre-interviews and focus groups before the creative workshops and after the eighth interview for the interviews conducted after the creative writing workshops. The number of participants allowed for data saturation.(Green and Thorogood 2018) An independent researcher (AA) with experience in qualitative methods coded 43% of the raw data with the rate of coding agreement assessed at 92% and disagreements discussed. Coded quotes were then organized by theme and subtheme.

For peer review only

For peer review only - http://bmjopen.bmj.com/site/about/guidelines.xhtml

RESULTS

Before the creative writing workshops: transplant patients' perspectives on previous experiences of sharing self-narratives

1. Participants' characteristics

Twenty-two lung transplant recipients, five liver transplant recipients and four heart transplant recipients took part in focus groups and individual interviews before the creative writing workshops. Of these 31 participants, one lung transplant recipient did not complete the sociodemographic form. Among the remainder, 17 were female and 13 were male. They were in their fifties. The majority described themselves as White and 21 reported having a college or university level of education. Table 1 summarizes participants' characteristics.

2. Key moments during the transplantation journey and past experiences of peer support

Across various hospital settings, timings, and types of organ, the transplant experiences reported by our participants share four common "key moments": (i) being notified that they will need to undergo an organ transplant; (ii) receiving the call once an organ is available; (iii) the immediate post-transplant period; and (iv) becoming aware of one's mortality. Table 2 presents interviews excerpts.

3. Previous experiences of sharing self-narratives

Most participants had experiences of sharing their stories prior to attending our creative workshops, in either spoken form (e.g. in other groups or for an organization) or written form (e.g. a letter to their donor or a web platform). Participants reported that the content of their previous self-narratives focused on advice and encouragements, resulting in valorization of experiential knowledge. Most participants felt that their previous experiences of sharing stories had been beneficial to their audience, namely by making others feel less isolated in their journey. However, two participants indicated that previous experiences of sharing self-narratives had been emotionally difficult, due to the act of opening up to others and being confronted by their immediate reactions.

For peer review only - http://bmjopen.bmj.com/site/about/guidelines.xhtml

Most participants also had previous experiences of hearing other transplant patients' stories, in either spoken form (e.g., in other support groups or in the hospital setting) or written form (e.g., a web platform). Participants highlighted the following needs that were met through hearing others' self-narratives: i) access to experiential knowledge different from healthcare professionals' expertise; and ii) encouragement, hope and reassurance. However, it is worth noting that one participant reported feeling overwhelmed and "saturated" after bearing witness to "too many narratives". Table 3 presents interview excerpts.

For peer review only

Experiences of attending creative writing workshop

1. Participant characteristics

Nine lung transplant recipients, four liver transplant recipients and one heart transplant recipient took part in 17 individual interviews to document their experience of attending creative writing workshops. Eight of them were female and six were male. They were in their fifties and 13 were White. The majority had a post-secondary education level. The average time since the transplant was more than seven years. Table 1 summarizes the participants' characteristics.

2. Positive and negative aspects of the creative writing workshops

Participants enjoyed the creative writing workshops. They reported that the creative writing workshops provided a respectful environment, with encouraging, open-minded, and qualified facilitators. Many participants conveyed that the creative writing workshops allowed them to reflect and gain better insight on their transplantation journey. The group setting was also reported as a notable strength of the workshops, providing a space for peer support and a feeling of connection to others. Many participants also highlighted that the workshops were stimulating and inspired them to invest in their creative process. Importantly, several participants reported that their creative writings had a positive effect on them by enabling self-expression and, more specifically, emotional expression. Some participants also mentioned that the workshops strengthened their understanding and appreciation of organ donation. Of note, one

For peer review only - http://bmjopen.bmj.com/site/about/guidelines.xhtml

participant who had undergone organ transplant several years prior to workshops emphasized that their primary interest in attending the workshops at the present time was to have a space for creativity that remains separate from their transplantation experience per se. Similarly, another participant indicated that they were attracted by the “opportunity to break free from the ‘sick talk’ and to instead delve into the arts”. Most participants would recommend the creative workshops to others who have undergone or are awaiting an organ transplant procedure.

The potential benefits of the creative writing workshops were clearly recognized by most participants. There was, however, a noteworthy heterogeneity regarding the participants’ experiences of the creative aspect of the workshops. While some participants reported feeling comfortable with the framework of the creative workshops, several of them reported feeling underqualified and intimidated by others’ artistic talents, with two participants describing a competitive environment among participants. Six participants expected the workshops to focus on the organ transplant experience specifically, and so they were disappointed by their perceived lack of testimonials through others’ artworks. In terms of logistics, participants commented on the length of the sessions and on the virtual setting. Two participants expressed that they would have liked more time for the group to share their creative writings, but one maintained that the sessions felt “lengthy” enough as they were. Moreover, some participants commented on the barriers to fluid communication with other participants when using a virtual platform such as Zoom: for example, the lack of nonverbal cues, the lack of eye contact, and the technical difficulties. This may at least partly account for the five participants who had the impression that others took either too much or too little space during the workshops, which made them feel less comfortable than they would have liked. Table 4 presents interview excerpts.

3. Therapeutic aspects of creative writing workshops and the associated peer support

For peer review only - http://bmjopen.bmj.com/site/about/guidelines.xhtml

The most frequently reported benefit of the creative process was its "therapeutic" effect, especially through the meaning-making that results from conceiving and sharing their creative writing. Almost half of the participants reported being pleased to discover and experiment with new ways of creating during the workshops, which resulted for some in the exploration of a different perspective of their own selves. Several participants also stressed that the creative format of the workshops was crucial to the voicing of their self-narratives and associated emotions. The elaboration and subsequent sharing of their self-narratives made participants feel "proud", "listened to", and "valued as a human being". Some participants also understood the creation of their artworks as a way to pay tribute and to value every stakeholder involved in the process of organ donation.

Participants who attended the creative writing workshops also reported "feeling connected to others". Workshops were viewed as a way to break isolation and as a source of peer support. Several participants noticed that the positive experience of sharing their creative writings with other participants during the workshops empowered them to be vulnerable in a similar way with their spouse or family members. This feeling of belonging within a group or a community was the cornerstone of various other benefits, such as sharing of experiential knowledge, being touched by others' narratives, putting into perspective and normalizing the participants' experiences, modelling resilience among participants, and cultivating open-mindedness and care.

While most of the participants' feedback highlighted beneficial aspects of peer support, some participants acknowledged that to bear witness to testimonials that were at times "darker" could be difficult, or even "triggering". Two participants noted the importance of "detachment" to avoid feeling overly distressed by others' testimonials. Yet, one participant reported "detachment" as an unwanted feeling which emerged upon discerning self-pity in others' testimonials. Table 5 presents interview excerpts.

For peer review only - http://bmjopen.bmj.com/site/about/guidelines.xhtml

DISCUSSION

This is the first study to examine heart, lung and liver transplant recipients' perspectives on the impact of creative writing workshops on their transplantation journey. We have previously reported the perspectives of kidney transplant candidates, kidney transplant recipients, and living donors on creative writing workshops.(Laneuville et al. 2022) However, heart, liver and lung transplant recipients face different challenges than kidney patients do, since transplantation is a life-saving treatment for them—there is no other option, in the way that dialysis represents an option for patients with end-stage kidney disease. Heart, liver and lung transplant candidates have to face the possibility of their mortality if a transplant is not offered, and also if there are serious adverse effects after transplantation. Becoming aware of one's own mortality was a theme reported by participants in our current study, whereas it was not reported by the kidney transplant candidates and kidney transplant recipients in our earlier study.

As reported by kidney transplant patients, the call for a transplant was a key moment in their transplantation journey. In recent studies conducted with heart and lung transplant patients, the call for transplant was associated with fear and anxiety.(Mestres-Soler et al. 2023, Stubber and Kirkman 2020) The participants in our study also highlighted the immediate post-transplantation journey. Such experience aligns with the dominant narrative of transplantation, where the transplantation itself is a healing event. This is a linear and restorative narrative where transplantation will allow patients to return to health and have a second chance.(Wasson 2021) (Mestres-Soler et al. 2023)

For the participants in our study, sharing stories or hearing other patients' stories during their transplantation journey were described as valuable peer support, and were viewed as a way to normalize their own experiences and put them into perspective. Also, sharing the same experiences of organ transplantation could make the listener more empathic and understanding than someone who has not

For peer review only - http://bmjopen.bmj.com/site/about/guidelines.xhtml

gone through the transplant experience.(Hsieh 2004) Moreover, sharing stories and listening to other patients' stories could help to better understand their identities as transplant patients but also as an individual. In this sense, peer support is a way of acquiring experiential knowledge.(Nilsson et al. 2022) In a previous study, it was even stated that only someone who has gone through the process could understand what heart transplantation is.(Mauthner et al. 2012) However, some patients did not want to be exposed to other patients' stories because they believed that each patient's experience is unique and so did not want to be scared by other patients' bad experiences. This aligns with the results of other studies showing that peer support could be valuable for people awaiting a heart or heart/lung transplantation because peers could provide hope, first-hand information and reassurance. However, it was also reported that some patients did not want to hear about other patients' stories because they did not want be scared or anxious about the future. (Stubber and Kirkman 2020)

Previous studies have also examined the role of illness narratives and storytelling that create sense and coherence in the illness experience and serve as a tool for coping.(Lanis et al. 2022, Rushforth et al. 2021) Moreover, when patients share their stories with an audience, the act of storytelling lets them negotiate the meanings of the stories and redefine their identities.(Hsieh 2004) A recent study conducted with liver transplant recipients used the creation of short videos and writing exercises during workshops in order to develop a digital story.(Wong-Mersereau et al. 2023) These workshops allow patients to expand their narrative possibilities and go beyond the archetypes associated with organ transplantation. Sharing the digital stories and commenting on other digital stories and videos allow participants in these workshops to create a sense of community and belonging.

For many participants, the creative writing workshops were described as "therapeutic" and made them feel valued as holistic human beings. This could be explained partly by the peer support and partly by the fact that workshops allowed vulnerable patients to break their isolation during the anxiety-inducing

For peer review only - http://bmjopen.bmj.com/site/about/guidelines.xhtml

period of the pandemics. That being said, the therapeutic quality of writing, for both mental and physical health, has been described previously by Pennebaker.(Pennebaker 2000b) He developed a technique of expressive writing that included the following principles: i) the writer openly acknowledges and accepts their emotions; ii) they become able to express those emotions; iii) they construct a meaningful story; and iv) writing provides a space for increased self-reflection.(Ruini and Mortara 2022) Given the standardized frame of expressive writing, it is distinct from creative writing where participants are encouraged to use different literary styles and use fiction and imagination in their writings. It is nonetheless striking to note the resemblance between the therapeutic elements described by our own participants and those reported by Pennebaker. A possible explanation is that expressive writing could be understood as a form of creative writing, hence resulting in similar cognitive and emotional processes being activated and similar therapeutic outcomes. A randomized controlled trial comparing expressive writing to neutral writing found peer support to be beneficial post-transplant only if there had been previous self-reflection and emotional expression.(Rini et al. 2014) We may therefore hypothesize that emotional expression during our creative workshops was central to their potentially therapeutic yield.

Advances in the field of organ transplantation over the past decades have resulted in considerable improvement in post-transplant survival rates.(Meyer 2018) However, organ transplantation is associated with existential challenges such as disruption in the sense of identity or selfhood. This disruption could be related to the alienation related to the disease leading to transplantation and the post-transplant care; to the symbolic nature of the transplanted organ, such as the heart; or to the bodily disruption, and the feeling of interconnectedness with the person who donated the organs.(Mauthner et al. 2015, Svenaeus 2012) Sharing narratives and peer support between transplant recipients was viewed by the participants as a way to normalize the transplantation journey and the changes of identity that could occur.

For peer review only - http://bmjopen.bmj.com/site/about/guidelines.xhtml

Limitations

There are some limitations related to our study sample. All our participants came from two urban transplant centres in Quebec. These participants, both before and after the creative writing workshops, were mainly of White ethnicity with a college or university degree, and a mean age of approximately 60 years. Because the creative writing workshops were conducted in French, we were not able to reach out to a greater diversity of patient ethnicities with a lower level of French literacy. The timing of the workshops, which were at night, could have been a barrier for some participants. The required time commitment may suit older, retired participants rather than young, working adults. The virtual format of the workshops, delivered over Zoom during the pandemic, also favoured patients with a stable or available Internet connection and digital literacy.

Another limitation in our data collection arises from the specificity of the multiple processes involved in the creative writing workshops. Although we were attentive to the reported experience of the creative writing process itself, most of the time the participants' answers did not permit a clear distinction between the action of writing and that of sharing one's writing and self-narratives in the workshop; the two actions were confounded together. Further studies are needed to tease out these different processes in relation to their reported therapeutic effects.

For peer review only

For peer review only - http://bmjopen.bmj.com/site/about/guidelines.xhtml

Conclusion

In conclusion, our creative workshops provided a space dedicated to individual creative expression as well as the sharing of self-narratives and peer support. Our participants described the creative process as therapeutic, possibly as a result of increased insight, meaning-making, emotional expression, and the feeling of being valued as a human being. Sharing of self-narratives and peer support were also found to be beneficial due to the feeling of connection with others, the sharing of experiential knowledge, and the normalization of experiences. Creative workshops that combine creative self-expression and peer support were found to be a feasible and beneficial intervention among transplant patients. More research is needed for the sake of studying the impacts of creative writing as an arts-based intervention, as opposed to other forms of storytelling: non-artistic (e.g. digital storytelling, life review and reminiscence therapy),(Wood, Waterman, and Pines 2021, Rios Rincon et al. 2022, King 2018) or psychotherapeutic (e.g. expressive writing).(Blechinger and Klosinski 2011, Costa and Abreu 2018, Pennebaker 2000a, Smyth and Pennebaker 2008)

For peer review only

For peer review only - http://bmjopen.bmj.com/site/about/guidelines.xhtml

Authorship

1. Laurence Ducharme participated in the data analysis and in the writing of the manuscript.
2. Marie-Françoise Malo participated in the conduct of the research and the data analysis, and in the writing of the manuscript.
3. Laurence Laneuville participated in the data analysis and in the writing of the manuscript.
4. Fabian Ballesteros participated in the conduct of the research and the data analysis, and in the writing of the manuscript.
5. Alyia Affdal participated in the conduct of the research and the data analysis, and in the writing of the manuscript.
6. Léonore Brassard participated in the conduct of the research and in the writing of the manuscript.
7. Benjamin Gagnon-Chainey participated in the conduct of the research and in the writing of the manuscript.
8. Pascale Millot participated in the conduct of the research and in the writing of the manuscript.
9. Catherine Mavrikakis participated in the research design, in the conduct of the research and the data analysis, and in the writing of the manuscript.
10. Simon Harel participated in the research design, in the conduct of the research and the data analysis, and in the writing of the manuscript.
11. Marie-Chantal Fortin participated in the research design, in the conduct of the research and the data analysis, and in the writing of the manuscript.

Data sharing Statement: Data will be available upon reasonable request to the corresponding author.

For peer review only

Table 1. Characteristics of participants in pre-workshop and post-workshop interviews

Characteristics	Pre-worshop[1] N=31 (%)	Post-Workshop N=14 (%)
**Type of organ transplant**		
Lung transplant recipients	22 (71)	9 (64)
Liver transplant recipients	5 (16)	4 (29)
Heart transplant recipients	4 (13)	1 (7)
**Sex**		
Female/male/unknown	17(55) /13 (42) /1 (3)	8 (57)/6 (43)/(0)
**Age (years)**		
Mean ±SD	54.9 +/-12.9<br>Range 29-72	55.1 +/-15.9<br>Range 26-73
**Race**		
White	27 (87)	13 (93)
Other	2(6)	0 (0)
Prefer not to answer	1(3)	1 (7)
Unknown	1(3)	**0 (0)**
**Employment status**		
Retired	10 (32)	6 (43)
Employed/self-employed	15 (48)	6 (43)
Unemployed	5 (16)	1 (7)
Unknown	1 (3)	1 (7)
**Level of education**		
College or university	21(68)	8 (57)
High school	8 (26)	3 (21)
Elementary school	1 (3)	2 (14)
Unknown	1 (3)	1 (7)
**Annual family income**		
More than $100K	5 (16)	4 (29)
$50K to $99,999	13 (42)	3 (21)
Less than $50K	7 (23)	5 (36)
Prefer not to answer	5 (16)	2 (14)
Unknown	1 (3)	0 (0)
**Time since transplant** (months ±SD)	114.3 +/-78.6<br>Range from 29-396	87.7 +/-57.9<br>**Range from** 9-188

1. Only 30 participants completed the socio-demographic questionnaire

For peer review only - http://bmjopen.bmj.com/site/about/guidelines.xhtml

TABLE 2: KEY MOMENTS IN THE TRANSPLANTATION JOURNEY

Themes and interview excerpts	N=31
**Being notified that they will need to undergo an organ transplant**<br><br>*"The most shocking moment, well, I think it was the moment when I was told that my liver was no longer good and that I was going to die, and that I was going to be on a waiting list for 6 months."* (male liver recipient)<br><br>*"It was when they told me that it was taking hold of me, that it was really taking hold of my heart. It's gives you...it gives you a bit of a shock. In the end, when you're faced with a fait accompli, the question is whether to let go and die or accept the heart."* (male heart recipient)	**5**
**Receiving the call once an organ is available**<br><br>*"...I got the phone call on the Monday, two days beforehand, saying I had to go in. You're afraid, but at the same time you're so happy, because for me it was just a matter of months then. She told me that it wasn't a matter of years anymore, so we were really looking forward to it."* (female liver recipient)<br><br>*"But it's really true that the call that came that night, well, I was called in the evening then I had to go into the hospital the next morning, but I think it was the evening, I would say it was the worst night of my life because you're saying to yourself, well tomorrow it's make or break, and you are hyper-aware of everything and the hours become very long. At the same time it goes by fast, but at the same time it feels long. You want to be positive and say to yourself, I'm going to come back, I'm going to come back in a few weeks, but at the same time you can't ignore the fact that the risk is there and that these are major surgeries. So I think that that was one of the heaviest experiences in my life."* (female lung recipient)	**5**
**The immediate post-transplant period**<br>*"One moment I remember was when I was all alone in my hospital room and it had been maybe 2 or 3 days, I don't know, a few days since my transplant, and then I was very, very vulnerable emotionally and then I thought about my donor and his family and it brought tears to my eyes. I was, it was mixed emotions because I was so happy, so grateful. I still am but I mean I was so like touched by the gesture, I was so like: Ah my god, it's true, I've got my lungs now. There."* (female lung recipient)<br><br>*"Then after that, there was a kind of euphoria that set in once I'd been transplanted, woken up, everything was fine. In the weeks that followed, I remember I was laughing so hard I couldn't stop, I was in a state of euphoria."* (female lung recipient)	**5**
**Becoming aware of one's mortality**<br>*"There were times when I really thought I was going to die, and now I'm having recurring nightmares. It's been three and a half years now, and it's left a real mark on me, but it's not so much a positive thing as a big change. I don't know."* (female lungs recipient)<br><br>*"Oh my God!" (Hesitations) "There are a few. I think the most memorable moment was one morning when I woke up at home and I was having a pulmonary embolism and thought I was going to die." (Sobbing)* (female lung recipient)	**6**

For peer review only - http://bmjopen.bmj.com/site/about/guidelines.xhtml

TABLE 3 – EXPERIENCES OF SHARING AND HEARING TRANSPLANT SELF-NARRATIVES BEFORE THE WORKSHOPS

Interview excerpts	N=31
**Prior experience of sharing their own stories with other people**	
**1. Self-narratives are a source of advice and information for other patients**	7
"People want to know how it's gonna go, if you have any tips or tricks for them or if there's anything they should know. They want to know about your experience, how things went for you." (female liver recipient)	
"I encouraged several others to go through with the transplant. They would say, no, it's too difficult, but I would tell them to take it one day at a time, they didn't have a choice. It's either a transplant or you die. You don't have a choice. You're better off accepting the transplant right off the bat." (female lung recipient)	
**2. Sharing transplant stories are a way to break the feeling of loneliness**	
"I think they feel less alone because even if you're surrounded by family, sometimes family doesn't truly understand what you're going through." (female liver recipient)	12
**3. Sharing transplant stories could have negative impact**	
"One person told me that I had scared them." (male lung recipient)	3
"I'd say that like the person I'd met face to face with her mother, well I saw that it had helped her a lot, but when I went back home, I had, it was like an emotional burden that was kind of heavy to bear. I'd found it a bit difficult there as it's still quite draining, we can definitely say. I think it's maybe not so for everyone, how can I put it? To open up so much here." (female, lung recipient)	
"I got angry because I felt that, at least for me, we're all different, and then the person was waiting for a transplant, and then she was, I felt that she was kind of disrespectful toward the transplant request, and then I just stopped talking to her because it just made me angry and then it just made me mad to see that there was like no respect toward the donor afterwards when he had his transplant there." (female lung recipient)	
**Prior experience of hearing other patients' stories**	
**1. Access to experiential knowledge**	11
"People want to know what's going to happen. People want to know how it's going to be, people want to know do you have stuff, is there anything I should know? They want to know about your experience, how it went for you too." (female liver recipient)	
"She told me about her journey, her transplant. She too had a rather difficult transplant, and today she was well, she had a girlfriend and then her girlfriend had a child. So because there was that situation as well, there was a worry if I'm going to be immunosuppressed while living with a child who brings all the germs from daycare. So to have that reference and then she'd say: You just need to wash your hands more. It's going to be all right then." (female lung recipient)	
**2. Encouragement, hope and reassurance**	
"It encouraged me... We all know that after a transplant, you're a bit worried about the follow-up and what can happen, and when I saw that she was blossoming and that she had	22

For peer review only - http://bmjopen.bmj.com/site/about/guidelines.xhtml

resumed her youthful life, because she was also 20 years old, yes, it encouraged me." (female lung recipient)<br><br>*"It was very encouraging to see how well she was doing. So that helped me a lot. ...So I'd say what helped me get through it the most was to see... Well maybe to be aware or to meet people who have great stories there, great successes there. And when I say a great success, it's important to be aware that there's a lot of adapting to do afterwards, and then sometimes there are other little problems that arise, but the fact remains that people can resume their activities and continue to live as normal a life as possible, despite certain limitations."* (female lung recipient)<br><br>*"Well, all of their stories, they've all left their mark on me. Each person is different there. It was all positive, really more the hope that was there because there's no hope when you're waiting for lungs. I had a month to live, so I couldn't wait for it to happen. It's fun to see the positive results."* (female lung recipient)	

For peer review only

For peer review only - http://bmjopen.bmj.com/site/about/guidelines.xhtml

TABLE 4: POSITIVE AND NEGATIVE ASPECTS OF THE CREATIVE WRITING WORKSHOPS

Interview excerpts	N=17
**Positive aspects of creative writing workshops**<br><br>*"There were some good discussions and even between the participants, it was very respectful and encouraging."* (female lung recipient)<br><br>*"It's the type of workshop that allows you to share, to learn, to broaden your outlook and get different perspectives on what others have gone through versus what we have gone through ourselves, all while exploring different facets of art. Whether through writing or other media, being able to participate and chat with others and see what they think builds community."* (female lung recipient)<br><br>*"I liked how open and respectful the workshop atmosphere was. I also liked that, in a more general context, it was okay to participate if we wanted to, but also to hold back at times if it felt better. I liked the format: there was good discussion, everyone could participate, be heard, and circle back on what was created."* (female lung recipient)	14
**Negative aspects of the creative writing workshops**<br><br>*"I found it a bit strange that many participants said absolutely nothing and just looked at the zoom. Maybe it's something I'm uncomfortable with because I don't use zoom very often, so yes, it was strange for me to see other participants watching, but saying absolutely nothing."* (female liver recipient)<br><br>*"I don't know why, when we were sharing our creations, I felt like it was such a personal thing. Maybe I'm getting it wrong and I don't want to... but it seemed that others were trying to impress or that there was some kind of contest to see who was the sickest. I don't know how to put it. I don't want to speak badly about anyone, it was just the impression I got, that it was a contest to see who had it the hardest."* (female lung recipient)<br><br>*"I think it's a bit of a shame that during the week when we went over everyone's artwork, at times there were too many of us for everyone to share in the same room, so we had to split into two groups and then we didn't get to see everyone's results. It was a bit sad, because it's interesting to see everyone's artwork."* (female liver recipient)	10

.

For peer review only - http://bmjopen.bmj.com/site/about/guidelines.xhtml

TABLE 5: IMPACT OF THE CREATIVE WRITING WORKSHOPS AND PEER SUPPORT

Interview excerpts	N=31
**1.Therapeutic effect of the creative writing and sharing them**	
"Sharing some pretty personal things allowed me to get rid of some feelings of guilt and it was the first time that I had spoken about certain subjects aloud, especially with people I didn't know, and it helped me, because I didn't feel judged." (female liver recipient)<br><br>*"I think it helps you make sense of what you've gone through. It helps you put it into words, because not everyone has the same vocabulary or can express themselves easily. Sometimes, hearing others talk about something turns on a light bulb inside you and you're like: Aha! That's true, I've never thought about that or considered it quite like that before. So I think that even if it didn't change anything, it helped me to make some sense of the different things that I've been through."* (female lung recipient)	10
**2. Exploration of different perspectives of their own selves**	10
"I got an interesting writing experience out of it. I'm pretty shy, so writing allowed me to explore certain paths. At the time of the workshops, I was feeling emotional overload, so I closed up. All in all, I'm satisfied with the experience." (male lung recipient)<br><br>*"I've spent my life in hospitals. I didn't want my illness to define me, but I really started to feel that that's what was happening. I'm more than my illness."* (female lung recipient)	
**3. Sharing creative writing is a source of pride**	8
"I think it helped me realize that every workshop helped me step outside of my comfort zone. All the creative writing, which is really not my forte, was a challenge, and that's what I liked about it." (male lung recipient)	
**4. Creative writing is a way to give back**	2
"It's something that's between us and also the fact that we have someone else's organ inside us, we have a part of someone else within us and it's also important to value this person who gave us life, it's thanks to this person who donated their organ or to their family. We're still here, we still exist, so it's really important to pay tribute to them, yes, in a way." (female liver recipient)<br><br>*"But I always have, not pleasure, but I like to talk about it and I've also done conferences for the Canadian Liver Foundation and then Partenaire Santé, and I find it so extraordinary that they managed to save my life and that I managed to have two transplants with two different livers in the space of 24 hours, that I'm always happy to be able to share that again because I think it's a feat that they accomplished."* (female liver recipient)	
**5. Creative writing workshops let one feel connected and alleviate loneliness**	5
"Every story is important, and hearing different stories helps you feel less alone, because transplantees aren't exactly common." (female liver recipient)<br><br>*"I recognized my own story and it was an eye-opener. We think we're unique, but after hearing those stories, we realize that we really aren't so unique. So, when someone says*	

For peer review only - http://bmjopen.bmj.com/site/about/guidelines.xhtml

something, I'll say: Ah! It's exactly the same for me. It doesn't bother me not to be so unique, it's actually comforting." (female lung recipient)	
**6. Sharing narratives with other empower patients to be vulnerable with others**	7
"Well, I was very moved by what people were experiencing, and I was also moved by the perception they had of the two artworks I presented, and they understood the emotions I was conveying through them.... And then it was time to write the text, and I must admit that all the emotions came out at that point. ...because it took me three times because my voice was shaking and then I was very, very moved to tell my story." (female liver recipient)<br><br>*"It made me realize that I wasn't the only one in much the same situation, and that we'd all been through difficult things for most of us, and that we're all still here, still seeing our doctors, still having our treatments, but it's like we're going, we're not giving up. It's hard to see that there are all kinds of different situations, but they're all difficult for everyone, depending on their circumstances. That's it, I think it made me realize that a lot. "* (female liver recipient)	
**7. Darker side of hearing other narratives**<br>*"Sometimes it can be upsetting to meet others who, like us, are sick and have the same chronic illnesses and challenges. The whole mental health approach kind of surprises me. I'm quite shocked because there are some pretty depressed people in the group, even though it's a different approach."* (female liver recipient)	3

For peer review only

For peer review only - http://bmjopen.bmj.com/site/about/guidelines.xhtml

REFERENCES

Anderson, S. M., J. Wray, A. Ralph, H. Spencer, T. Lunnon-Wood, and K. Gannon. 2017. "Experiences of adolescent lung transplant recipients: A qualitative study." *Pediatr Transplant* 21 (3). doi: 10.1111/petr.12878.

Beilby, S., R. Moss-Morris, and L. Painter. 2003. "Quality of life before and after heart, lung and liver transplantation." *N Z Med J* 116 (1171):U381.

Blechinger, T., and G. Klosinski. 2011. "[The meaning of bibliotherapy and expressive writing in child and adolescent psychiatry]." *Prax Kinderpsychol Kinderpsychiatr* 60 (2):109-24. doi: 10.13109/prkk.2011.60.2.109.

Bruce, A., H. Daudt, and S. Breiddal. 2018. "Can Writing and Storytelling Foster Self-care?: A Qualitative Inquiry Into Facilitated Dinners." *J Hosp Palliat Nurs* 20 (6):554-560. doi: 10.1097/njh.0000000000000478.

Cierpka, A., K. Małek, and M. Horodeńska. 2015. "What does it mean to live after heart transplantation? The lived experience of heart transplant recipients. A qualitative study." *Health Psychology Report.* 3 (2). doi: 10.5114/hpr.2015.49678.

Costa, AC, and MV. Abreu. 2018. "Expressive and creative writing in the therapeutic context: From the different concepts to the development of writing therapy programs." *Psychologica* 61 (1):69-86.

Currier, H., and C. T. Zimmerman. 2019. "Use of Creative Writing to Illustrate Lived Experiences in Hemodialysis-Dependent Children with Chronic Kidney Failure." *Nephrol Nurs J* 46 (3):293-336.

Dew, M. A., and A. F. DiMartini. 2005. "Psychological disorders and distress after adult cardiothoracic transplantation." *J Cardiovasc Nurs* 20 (5 Suppl):S51-66. doi: 10.1097/00005082-200509001-00007.

Duffy, J. P., K. Kao, C. Y. Ko, D. G. Farmer, S. V. McDiarmid, J. C. Hong, R. S. Venick, S. Feist, L. Goldstein, S. Saab, J. R. Hiatt, and R. W. Busuttil. 2010. "Long-term patient outcome and quality of life after liver transplantation: analysis of 20-year survivors." *Ann Surg* 252 (4):652-61. doi: 10.1097/SLA.0b013e3181f5f23a.

Forzoni, S., M. Perez, A. Martignetti, and S. Crispino. 2010. "Art therapy with cancer patients during chemotherapy sessions: an analysis of the patients' perception of helpfulness." *Palliat Support Care* 8 (1):41-8. doi: 10.1017/s1478951509990691.

Green, J., and N. Thorogood. 2014. *Qualitative methods for health research*. Third Ed ed. London: SAGE Publiications Ltd.

Green, Judith, and Nicki Thorogood. 2018. *Qualitative Methods for Health Resaerch*. Fourth Edition ed. Thousand Oaks, California: Sage publishing.

Ho, E. W., A. L. Murillo, L. A. Davis, Y. A. Iraheta, S. M. Advani, A. Feinsinger, and A. D. Waterman. 2022. "Findings of living donation experiences shared on a digital storytelling platform: A thematic analysis." *PEC Innov* 1:100023. doi: 10.1016/j.pecinn.2022.100023.

Hsieh, Elaine. 2004. "Stories in Action and the Dialogic Management of Identities: Storytelling in Transplant Support Group Meetings." *Research on Language and Social Interaction* 37:39-70.

Kelly, M. P., and H. Dickinson. 1997. "The Narrative Self in Autobiographical Accounts of Illness." *The Sociological Review* 45 (2).

King, K. D. 2018. "Bringing Creative Writing Instruction into Reminiscence Group Treatment." *Clin Gerontol* 41 (5):438-444. doi: 10.1080/07317115.2017.1405858.

Laneuville, L, F. Ballesteros Gallego, A. Affdal, M-F Malo, L Brassard, B Gagnon-Chainey, P Millot, S Harel, C Mavrikakis, and M-C Fortin. 2022. "Perspectives of Kidney Transplant Recipients,

For peer review only - http://bmjopen.bmj.com/site/about/guidelines.xhtml

Transplant Candidates and Living Kidney Donors on the Role of Patients' Self-narratives and Experiences of Creative Writing Workshops: A Qualitative Study." *Canadian Journal of Kidney Health and Disease* 9:1-17. doi: 10.1177/20543581221132742

Lanis, A., E. Tu, M. Peskin, M. Melendez, G. Tarshish, A. Akinsete, A. Hoffman, K. Kenney-Riley, T. Rubinstein, and D. Wahezi. 2022. "Storytelling of Young Adults with Chronic Rheumatologic Illnesses: A Pilot Study." *Healthcare (Basel)* 10 (10). doi: 10.3390/healthcare10101979.

Marini, M. G. 2016. *Narrative Medicine: Bridging the Gap between Evidence-Based Care and Medical Humanities*: Springer International Publishing.

Mauthner, O., E. De Luca, J. Poole, M. Gewarges, S. E. Abbey, M. Shildrick, and H. Ross. 2012. "Preparation and Support of Patients through the Transplant Process: Understanding the Recipients' Perspectives." *Nurs Res Pract* 2012:547312. doi: 10.1155/2012/547312.

Mauthner, O. E., E. De Luca, J. M. Poole, S. E. Abbey, M. Shildrick, M. Gewarges, and H. J. Ross. 2015. "Heart transplants: Identity disruption, bodily integrity and interconnectedness." *Health (London)* 19 (6):578-94. doi: 10.1177/1363459314560067.

Mestres-Soler, O., R. Gómez-Ibáñez, E. Zuriguel-Pérez, R. Escobar-Fornieles, M. Aguayo-González, J. M. Leyva-Moral, and C. E. Watson. 2023. "Expectations of patients awaiting lung transplantation: A qualitative study." *Nurs Open* 10 (6):3766-3773. doi: 10.1002/nop2.1634.

Meyer, K. C. 2018. "Recent advances in lung transplantation." *F1000Res* 7. doi: 10.12688/f1000research.15393.1.

Nilsson, K., M. Westas, G. Andersson, P. Johansson, and J. Lundgren. 2022. "Waiting for kidney transplantation from deceased donors: Experiences and support needs during the waiting time - A qualitative study." *Patient Educ Couns* 105 (7):2422-2428. doi: 10.1016/j.pec.2022.02.016.

Pennebaker, J. W. 2000a. "Telling stories: the health benefits of narrative." *Lit Med* 19 (1):3-18. doi: 10.1353/lm.2000.0011.

Pennebaker, JW. 2000b. "“Telling stories: the health benefits of narrative”." *Lit Med* 19 (1):3-18.

Rini, C., J. Austin, L. M. Wu, G. Winkel, H. Valdimarsdottir, A. L. Stanton, L. Isola, S. Rowley, and W. H. Redd. 2014. "Harnessing benefits of helping others: a randomized controlled trial testing expressive helping to address survivorship problems after hematopoietic stem cell transplant." *Health Psychol* 33 (12):1541-51. doi: 10.1037/hea0000024.

Rios Rincon, A. M., A. Miguel Cruz, C. Daum, N. Neubauer, A. Comeau, and L. Liu. 2022. "Digital Storytelling in Older Adults With Typical Aging, and With Mild Cognitive Impairment or Dementia: A Systematic Literature Review." *J Appl Gerontol* 41 (3):867-880. doi: 10.1177/07334648211015456.

Romm, K. L., O. Synnes, and H. Bondevik. 2022. "Creative writing as a means to recover from early psychosis- Experiences from a group intervention." *Arts Health*:1-14. doi: 10.1080/17533015.2022.2130379.

Rosenberger, E. M., A. F. DiMartini, A. J. DeVito Dabbs, C. A. Bermudez, J. M. Pilewski, Y. Toyoda, and M. A. Dew. 2016. "Psychiatric Predictors of Long-term Transplant-Related Outcomes in Lung Transplant Recipients." *Transplantation* 100 (1):239-47. doi: 10.1097/tp.0000000000000824.

Ruini, C., and C. C. Mortara. 2022. "Writing Technique Across Psychotherapies-From Traditional Expressive Writing to New Positive Psychology Interventions: A Narrative Review." *J Contemp Psychother* 52 (1):23-34. doi: 10.1007/s10879-021-09520-9.

Rushforth, A., E. Ladds, S. Wieringa, S. Taylor, L. Husain, and T. Greenhalgh. 2021. "Long Covid - The illness narratives." *Soc Sci Med* 286:114326. doi: 10.1016/j.socscimed.2021.114326.

Sandelowski, M. 2000. "Whatever happened to qualitative description?" *Res Nurs Health* 23 (4):334-40. doi: 10.1002/1098-240x(200008)23:4<334::aid-nur9>3.0.co;2-g.

Sandelowski, M. 2010. "What's in a name? Qualitative description revisited." *Res Nurs Health* 33 (1):77-84. doi: 10.1002/nur.20362.

For peer review only

For peer review only - http://bmjopen.bmj.com/site/about/guidelines.xhtml

Smyth, J. M., and J. W. Pennebaker. 2008. "Exploring the boundary conditions of expressive writing: In search of the right recipe." *Br J Health Psychol* 13 (Pt 1):1-7. doi: 10.1348/135910707X260117.

Stubber, C., and M. Kirkman. 2020. "The experiences of adult heart, lung, and heart-lung transplantation recipients: A systematic review of qualitative research evidence." *PLoS One* 15 (11):e0241570. doi: 10.1371/journal.pone.0241570.

Svenaeus, F. 2012. "Organ transplantation and personal identity: how does loss and change of organs affect the self?" *J Med Philos* 37 (2):139-58.

Synnes, O., K. L. Romm, and H. Bondevik. 2021. "The poetics of vulnerability: creative writing among young adults in treatment for psychosis in light of Ricoeur's and Kristeva's philosophy of language and subjectivity." *Med Health Care Philos* 24 (2):173-187. doi: 10.1007/s11019-020-09998-5.

Tong, A., P. Sainsbury, and J. Craig. 2007. "Consolidated criteria for reporting qualitative research (COREQ): a 32-item checklist for interviews and focus groups." *Int J Qual Health Care* 19 (6):349-57. doi: 10.1093/intqhc/mzm042.

Wasson, S. 2021. "Waiting, strange: transplant recipient experience, medical time and queer/crip temporalities." *Med Humanit* 47 (4):447-455. doi: 10.1136/medhum-2021-012141.

Wong-Mersereau, Chloe, Fraser Allen Best, Lia Tarachansky, Shabnam Sukhdev, Mary Bunch, Alexandra Frankel, Brad Necyk, Kelly Fritsch , and Suze Berkhout. 2023. "Layered Methodologies: Innovating Multimodal Qualitative Research in Liver Transplantation." *Journal of Liver Transplantation* 12:1-10.

Wood, E. H., A. D. Waterman, and R. Pines. 2021. "Storytelling to Inspire Dialysis Patients to Learn about Living Donor Kidney Transplant." *Blood Purif* 50 (4-5):655-661. doi: 10.1159/000512651.

For peer review only

Figure 1 – Flowchart for the recruitment process

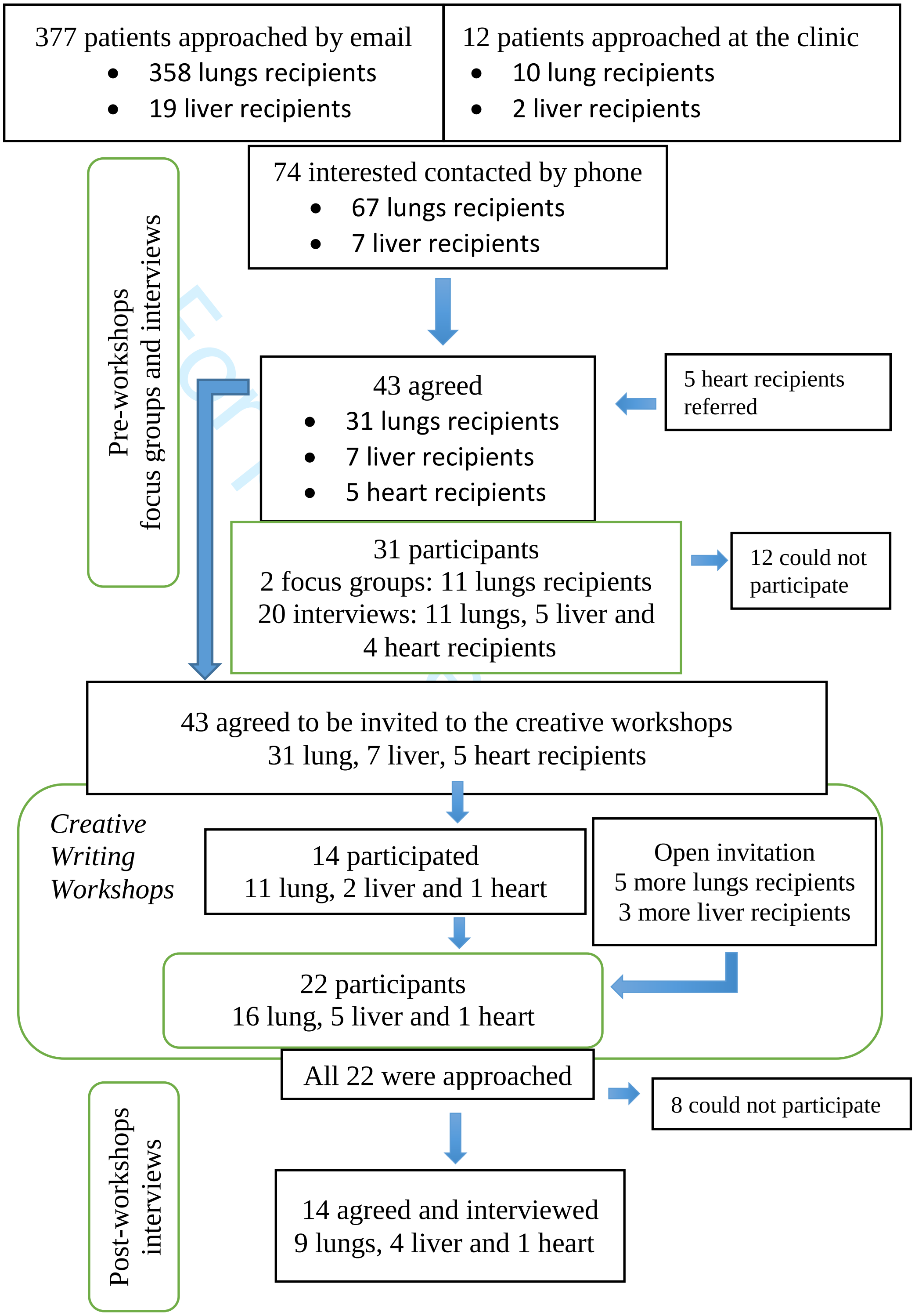

Quebec Lung, Liver, and Heart Transplant Recipients' Perspectives on Self-Narratives and Their Experiences in Creative Writing Workshops During the Transplantation Journey: A Qualitative Study

Journal:	*BMJ Open*
Manuscript ID	bmjopen-2024-096791.R1
Article Type:	Original research
Date Submitted by the Author:	05-Nov-2025
Complete List of Authors:	Ducharme, Laurence; Université de Montréal Faculté de Médecine<br>Malo, Marie-Françoise; Université de Montréal, École de santé publique, Bioethics Department<br>Affdal, Aliya; Université de Montréal, École de santé publique, Bioethics Department<br>Ballesteros, Fabian; Centre Hospitalier de l'Université de Montréal<br>Laneuville, Laurence; Université de Montréal Faculté de Médecine, Département de psychiatrie et d'addictologie,<br>Brassard, Leonore; Université de Montréal, Chaire McConnell-Université de Montréal sur les récits de don et de la vie en contexte de soins; Université du Québec à Trois-Rivières, Département de lettres et communication sociale<br>Gagnon-Chainey, Benjamin; Université de Montréal, Chaire McConnell-Université de Montréal sur les récits de don et de la vie en contexte de soins<br>Millot, Pascale; Université de Montréal, Chaire McConnell-Université de Montréal sur les récits de don et de la vie en contexte de soins<br>Mavrikakis, Catherine; Université de Montréal, Chaire McConnell-Université de Montréal sur les récits de don et de la vie en contexte de soins; Université de Montréal Faculté des Arts et des Sciences, Département des littératures de langues françaises<br>Harel, Simon; Université de Montréal, Chaire McConnell-Université de Montréal sur les récits de don et de la vie en contexte de soins; Université de Montréal Faculté des Arts et des Sciences, Département de littératures et de langues du monde<br>Fortin, Marie-Chantal; Université de Montréal Faculté de Médecine; Université de Montréal, École de santé publique, Bioethics Department; Centre Hospitalier de l'Université de Montréal; Université de Montréal, Chaire McConnell-Université de Montréal sur les récits de don et de la vie en contexte de soins
**Primary Subject Heading**:	Qualitative research
Secondary Subject Heading:	Patient-centred medicine, Qualitative research
Keywords:	TRANSPLANT MEDICINE, QUALITATIVE RESEARCH, Peer Group, Literature

I, the Submitting Author has the right to grant and does grant on behalf of all authors of the Work (as defined in the below author licence), an exclusive licence and/or a non-exclusive licence for contributions from authors who are: i) UK Crown employees; ii) where BMJ has agreed a CC-BY licence shall apply, and/or iii) in accordance with the terms applicable for US Federal Government officers or employees acting as part of their official duties; on a worldwide, perpetual, irrevocable, royalty-free basis to BMJ Publishing Group Ltd ("BMJ") its licensees and where the relevant Journal is co-owned by BMJ to the co-owners of the Journal, to publish the Work in this journal and any other BMJ products and to exploit all rights, as set out in our licence.

The Submitting Author accepts and understands that any supply made under these terms is made by BMJ to the Submitting Author unless you are acting as an employee on behalf of your employer or a postgraduate student of an affiliated institution which is paying any applicable article publishing charge ("APC") for Open Access articles. Where the Submitting Author wishes to make the Work available on an Open Access basis (and intends to pay the relevant APC), the terms of reuse of such Open Access shall be governed by a Creative Commons licence – details of these licences and which Creative Commons licence will apply to this Work are set out in our licence referred to above.

Other than as permitted in any relevant BMJ Author's Self Archiving Policies, I confirm this Work has not been accepted for publication elsewhere, is not being considered for publication elsewhere and does not duplicate material already published. I confirm all authors consent to publication of this Work and authorise the granting of this licence.

For peer review only

TITLE: Quebec Lung, Liver, and Heart Transplant Recipients' Perspectives on Self-Narratives and Their Experiences in Creative Writing Workshops During the Transplantation Journey: A Qualitative Study

AUTHORS: Laurence Ducharme, MD*, Marie-Françoise Malo, PhD(c)[2*,] Aliya Affdal, MSc[2,] , Fabian Ballesteros, MSc[3], Laurence Laneuville, MD, MA[4],Léonore Brassard, PhD[5], Benjamin Gagnon-Chainey, PhD[5], Pascale Millot, PhD(c)[5,6], Catherine Mavrikakis, PhD[5,6], Simon Harel, PhD[5,7], Marie-Chantal Fortin, MD, PhD [1,2,3,5,8]

1. Faculty of Medicine, Université de Montréal, Montréal, Canada
2. Bioethics Program, École de santé publique de l'Université de Montréal, Montréal, Canada
3. Centre de recherche du CHUM, Montréal, Canada
4. Faculty of Medicine, Département de psychiatrie et d'addictologie, Université de Montréal, Montréal, Canada
5. Chaire McConnell-Université de Montréal sur les récits de don et de la vie en contexte de soins, Université de Montréal, Montréal, Canada
6. Département des littératures de langues françaises, Faculté des arts et sciences, Université de Montréal, Montréal, Canada
7. Département de littératures et de langues du monde, Faculté des arts et sciences, Université de Montréal, Montréal, Canada
8. Canadian Donation and Transplantation Research Program, Edmonton, Canada

*"Laurence Ducharme and Marie-Françoise Malo have made equal contributions to this manuscript.

CORRESPONDING AUTHOR:
Marie-Chantal Fortin, MD, PhD, FRCPC
Centre de recherche du CHUM
900, rue St-Denis, Salle R12-418
Montréal (Québec) H2X 0A9
514-890-8000 ext. 25712
marie-chantal.fortin.med@ssss.gouv.qc.ca

WORD COUNT : 4,918

For peer review only

Abstract

Background: Although solid organ transplantation is a life-saving procedure, it is also associated with numerous challenges for patients. Creative writing has been described as a therapeutic tool for patients with chronic disease. Between 2020 and 2022, we held creative writing workshops with patients and professional writers. During these workshops, patients were invited to explore different aspects of their transplantation journey through different literary styles.

Objectives: The aim of this study is to understand lung, liver and heart transplant patients' perspectives on the role of patient self-narratives and creative writing workshops during their transplantation journey, and collect testimonies on their experiences in the workshops.

Design: Focus groups and individual interviews.

Setting: The Centre hospitalier de l'Université de Montréal (CHUM) lung and liver transplant programs and the Montreal Heart Institute (MHI) heart transplant program.

Participants: Lung, liver and heart transplant recipients attending the CHUM and the MHI transplant clinic between September 2020 and August 2022.

Methods: Before the creative writing workshop, we conducted two focus groups and individual interviews with 22 lung recipients, five liver transplant recipients and four heart transplant patients. After their participation in the creative workshop, we conducted 17 semi-structured interviews with nine lung transplant recipients, four liver transplant recipients and one heart transplant recipient (three participants participated twice in interviews). Interviews were transcribed for subsequent qualitative description analyses.

Results: Most participants had previous experiences of sharing their transplant stories and considered that a web-based platform where transplant patients share their stories could be highly beneficial for future transplant patients and the public. The creative workshops were described as "therapeutic" since they let participants express their emotions and realities. Peer support and connection to other people

For peer review only

contributed to normalize their transplant journey and helped participants put their experiences into perspective.

Conclusion: Creative writing workshops and peer support through the sharing of self-narratives are a feasible approach that participants have described as beneficial. Future studies are needed to assess the benefits of patients' creative writings on other patients and on caregivers.

For peer review only

For peer review only - http://bmjopen.bmj.com/site/about/guidelines.xhtml

Strengths and limitations:

- The qualitative design of our study allowed us to gain in-depth insights into participants' perspectives on self-narratives and their experiences in creative writing workshops.
- Including transplant recipients of different organs enriched our understanding of how self-narratives and creative writing workshops may support diverse patient experiences.
- Using both individual interviews and focus groups generated richer thematic content and enhanced the depth of our analysis.
- One limitation of our study is that most participants were White, highly educated, and recruited from three urban transplant programs, which may limit the generalizability of our findings.

Funding

This research project was funded by the Chaire McConnell-Université de Montréal sur les récits de don et de la vie en contexte de soins and by the Audace Program of the Fonds de recherche Québec. Dr. Marie-Chantal Fortin is a research scholar for the Fonds de recherche du Québec – Santé (FRQS) and an investigator for the Canadian Donation and Transplantation Research Program.

Competing Interests

Laurence Ducharme, Marie-Françoise Malo, Laurence Laneuville, Fabian Ballesteros, Alyia Affdal, Léonore Brassard, Benjamin Gagnon-Chainey, Pascale Millot, Catherine Mavrikakis, Simon Harel and Marie-Chantal Fortin have no competing interests to declare. The funders had no involvement in the study design; in the collection, analysis and interpretation of data; in the writing of the manuscript; and the decision to submit the paper for publication.

For peer review only - http://bmjopen.bmj.com/site/about/guidelines.xhtml

For peer review only

For peer review only - http://bmjopen.bmj.com/site/about/guidelines.xhtml

INTRODUCTION

Heart, lung and liver transplantations are treatments of choice for end-stage cardiac, lung or liver disease.[1] Living with a life-threatening illness leads to personal and social changes that can be difficult to cope with.[2 3] Despite reporting a better quality of life after transplantation,[4] emotional and psychological impacts of a transplant can remain long after the physical improvement of the recipient.[5 6]

Storytelling and art-based intervention (ABI) have gained a lot of traction in recent years as ways to bolster patients' recovery journey.[7] These approaches have been shown to benefit patients with mental illnesses,[8 9] cancer patients,[10] and patients with chronic illnesses.[11 12] ABIs can encompass several different art forms—from creative writing, to visual arts, to music.[8] Storytelling can help patients make sense of their emotions and can offer a setting and a framework for them to explore difficult aspect of their illness journey.[13] The subsequent dissemination of these stories may also help others during their own journey of care towards and after transplantation.[14].Our research group has already demonstrated, in previous work, the benefits of creative writing workshops and the diffusion of these self-narratives and creative writings for kidney transplant recipients, kidney transplant candidates and living kidney donors.[15] This study expands upon our earlier research by examining the perspectives of heart, lung, and liver transplant recipients on the role of creative writing and their experiences participating in creative writing workshops.

The objective of this study was twofold. First, prior to the creative writing workshops, transplant patients were invited to share their perspectives on the experience of narrating their transplantation journey. Second, patients who participated in the creative writing workshops were asked about their experiences in the workshops and the impact of sharing their self-narratives and creative writings. This study aims to

For peer review only

deepen understanding of the role that creative writing workshops and arts-based interventions can play in supporting lung, liver, and heart transplant recipients throughout their transplantation journey.

For peer review only

For peer review only - http://bmjopen.bmj.com/site/about/guidelines.xhtml

METHODS

This study was exploratory in nature and used semi-structured interviews and focus groups with heart, lung and liver transplant recipients. It employed the COREQ checklist for reporting qualitative research.[16] We conducted individual interviews and focus groups before participants attended the creative writing workshops, and individual interviews after the workshops. The CHUM institutional research ethics committee (CHUM CE20.206) approved the study; and all the participants gave written informed consent to take part in the pre-workshop focus groups and in the interviews held before and after the workshops. All recipients received organs from deceased donors, recovered with valid authorization and in accordance with Canadian legislation, international standards, and the ethical principles outlined in the Declaration of Istanbul on Organ Trafficking and Transplant Tourism. [17] After the workshops, there was a separate consent process regarding the decision to post the creative writings on the website anonymously or not.

Interviews and focus groups conducted before the creative writing workshops

Recruitment for focus groups and interviews was carried out between December 2020 and August 2022. Convenience sampling was the chosen approach for recruiting participants. French-speaking adults who were recipients of a heart, lung or liver transplant, and were followed at the two transplant clinics, were invited to participate by means of letters, posters, emails and flyers distributed at the transplant clinic. Directors of the lung and liver transplant programs sent an invitation email to 377 patients (358 lung recipients and 19 liver recipients), followed by a telephone call made by a member of the research team (FB or MFM). The heart transplant clinic approached the patients directly and obtained their informed consent before referring them to the research coordinator (FB).

Sixty-seven lung recipients and seven liver recipients agreed to receive more information about the study and were contacted by telephone by a member of the research team providing further details (FB). Of

For peer review only - http://bmjopen.bmj.com/site/about/guidelines.xhtml

these 74 patients, 38 accepted to participate and signed the informed consent (31 lung recipients and seven liver recipients). The heart transplant clinic referred five heart transplant recipients who had already consented to participate. From the group of 43 patients who had consented, 12 later declined to participate (nine lung recipients, two liver recipients and one heart recipient): 11 patients who had time constraints, and one who wanted to participate in person, which was impossible during the COVID pandemic. Participants received financial compensation for their time (CAN$30). Figure 1 summarizes the recruitment process.

The timing of the two focus groups was determined by the participants' and researchers' availability. For the participants who could not be scheduled for a focus group, we conducted an individual interview. During the focus groups, participants were welcomed by the research assistant who had contacted them (FB). The focus groups were facilitated by the principal investigator (MCF), who had experience in qualitative research and in conducting focus groups and was not involved in their transplant care. It was made clear from the beginning of the focus groups that whatever was addressed during the discussion would have no impact on their clinical care. The focus groups began with brief introductions of every participant, after which the research team made a short presentation on creative writing and medicine and explained the objectives of the study. Participants had an opportunity to ask questions about the presentation. Discussion then began and participants were invited to express their opinions and perspectives freely. The research assistant (FB), took notes during the focus groups. All the discussions were recorded and transcribed. One focus group lasted 60 minutes and the other one lasted 90 minutes. An individual interview was proposed for anyone who could not attend a focus group. Fifteen interviews were conducted by telephone and five by videoconference by a member of the research team (FB, AA and MFM). All the interviews were conducted in French. The interviews lasted around 30 minutes on average (ranging from 14 to 58 minutes) and were recorded and transcribed.

For peer review only - http://bmjopen.bmj.com/site/about/guidelines.xhtml

For peer review only

The issues covered during the interviews and focus groups were outlined in an interview guide with open-ended questions addressing the following topics: i) the most significant moment in the interviewee's transplantation journey; ii) experiences of sharing transplant self-narratives; iii) potential benefits or challenges of sharing self-narratives; iv) perspectives on publishing patients' self-narratives and creative writings on a web platform; and v) socio-demographic data. Given that no study has been conducted of patients' perspectives on sharing self-narratives pertaining to the transplantation journey, our interview guide was designed to elicit their experiences. The guide was developed by the research team from the literature on creative writing in medicine (MCF, CM, SH). Consistent with qualitative methodology, the interview guide was modified during the study as new topics emerged from the interviews.[16] (See Supplementary Material)

Description of creative writing workshops

Between September 2020 and April 2022, we conducted fourteen 90-minute virtual creative writing workshops with heart, lung, and liver transplant recipients. Each workshop was held two weeks apart and facilitated by two members of the research team with experience in workshop facilitation (SH and CM), along with one of fourteen guest artists. The artists represented a range of disciplines, including novel writing, poetry, comic art, puppetry, and screenwriting, with the goal of exposing participants to diverse artistic media and experiences. Each artist participated in a single workshop (i.e., two 90-minute sessions), while research team members were present throughout all sessions. Artists varied in their experience with ABIs, and most had not previously led ABIs with patients. Table 1 provides an overview of the titles (when applicable) and artistic disciplines of each intervention. Figure 2 presents a visual representation of the intervention, following the TIDieR checklist. [18]

The first session, which was more theoretical, introduced the theme and artistic medium, allowing participants to familiarize themselves with the art form. Instructions for the creative activity were also

For peer review only - http://bmjopen.bmj.com/site/about/guidelines.xhtml

provided. Participants then had two weeks to complete their creations. The second session served as a feedback forum, where participants could share their work, receive feedback, and discuss the meaning and message behind their creations, which often explored different aspects of their transplantation journey. Participants who consented were invited to publish their creative writing on the https://lorganon.ca website.

Although attendance was not restricted, participation averaged 8 to 10 individuals per workshop, fostering a small-group environment conducive to sharing and discussion. Transplant recipients were free to attend as many workshops as they wished. Each workshop invitation included information about the theme, artistic medium, and guest artist, and was distributed to a list of interested individuals. As a result, attendance varied: some participants attended all sessions, others joined only one, and some participated without actively sharing their artwork. No specific expectations were placed on participants, in keeping with the guiding principle that ABIs can engage both those who create art and those who experience it.[19]

Interviews conducted after the creative writing workshops

Forty-three of the people who consented to participate (30 lung transplant recipients, seven liver transplant recipients, five heart transplant recipient and one lung transplant candidate) agreed to receive an invitation to take part in the creative writing workshops, and 14 participated in at least one of the workshops (11 lung patients, two liver patients and one heart patient), which were held virtually between September 2020 and April 2022. Eight new transplant recipients (five lung recipients and three liver recipients) also joined the workshops (total of 22 participants: 16 lung recipients, five liver recipients and one heart recipient). All the participants (22) in the creative writing workshops were invited to be interviewed individually so that the team could gather their experiences. Nine lung transplant recipients, four liver transplant recipients and one heart transplant recipient agreed to participate and provided

For peer review only - http://bmjopen.bmj.com/site/about/guidelines.xhtml

informed consent. Two patients (one heart recipient and one liver recipient) were lost to follow-up and six lung transplant recipients chose not to participate because of their health condition.

Interviews were conducted between October 2021 and April 2022. Seventeen interviews were conducted. Thirteen interviews were conducted by telephone and four by videoconference by a member of the research team (AA). Participants received financial compensation for their time (CAN$20). It was deemed more appropriate to conduct individual interviews because of the reduced number of post-workshop participants, and for the sake of capturing their individual experience, rather than put them back into the same context as the workshops, where they possibly had pre-existing dynamics. The interviews lasted between 10 and 30 minutes (about 16 minutes on average) and were recorded and transcribed. The following topics were addressed during the interviews: i) experiences of participating in the creative writing workshops; ii) impact of the workshops and writing; iii) elements liked and disliked during the workshops; iii) experiences of sharing stories about their transplantation journey during the workshops; iv) how to post stories on a web platform; v) sociodemographic data. The interview guide was developed by the research team (MCF, CM, SH). (See supplementary material)

For peer review only

Data analysis

We used a qualitative description approach to describe and analyze transplant recipients' perspectives and experiences.[20] The goal of this pragmatic approach was to use thematic analysis [21] in order to remain as close as possible to the data collected during the interviews and provide a comprehensive summary of the topic studied.[22] The latest version of the NVivo (Lumivero) software facilitated the analysis. Initial coding frames based on the interview grid were developed by MFM, LD and AA. Codes were added and taken out based on the content of the interviews. The research team met frequently to discuss the coding frame and the data analysis. A member of the research team with expertise in qualitative methodology (MFM) coded the interviews and no new codes were created after the eleventh interview for the pre-interviews and focus groups before the creative workshops and after the eighth interview for the interviews conducted after the creative writing workshops.

During the coding process, a member of the research team who was new to qualitative work, but is both a medical and literary student (LD), used the coding grid to code two interviews in order to assess inter-coder reliability mid-way through coding. LD then met with the team to discuss the importance and relevance of codes to the study until consensus was reached. Because MFM participated in 9 of the 14 ABI workshops, this step was considered essential to help maintain neutrality in the coding process. Afterward, MFM re-coded the previously coded material using the modified coding grid. The research team further engaged in collaborative discussions to resolve any discrepancies in theme identification and refinement, fostering a consensus-driven approach.The number of participants allowed for data saturation.[23] At the end of the coding process, a team member (AA) with experience in qualitative methods, but who had not participated in the ABI workshops, coded a portion of the raw data—purposively selecting transcripts from multiple stages of the coding process—to ensure coding accuracy and consistency across the dataset. The team then discussed the results until intercoder agreement was reached. None of the coders had personal experience as a transplant patient or as a caregiver for a

For peer review only - http://bmjopen.bmj.com/site/about/guidelines.xhtml

transplant patient. However, both MFM and AA have worked for several years on a qualitative research team studying the lived experiences of transplant candidates, patients, and caregivers. Coded quotes were then organized by theme and subtheme.

Patient and public involvement

No patient partners were actively involved in this research project.

For peer review only

RESULTS

Before the creative writing workshops: transplant patients' perspectives on previous experiences of sharing self-narratives

1. Participants' characteristics

Eleven lung transplant recipients took part in two virtual focus groups: seven in the first and four in the second. Eleven lung transplant recipients, five liver transplant recipients and four heart transplant recipients took part in individual interviews before the creative writing workshops. Of these 31 participants, one lung transplant recipient did not complete the sociodemographic form. Among the remainder, 17 were female and 13 were male. They were in their fifties. The majority described themselves as White and 21 reported having a college or university level of education. Table 2 summarizes participants' characteristics.

2. Key moments during the transplantation journey and past experiences of peer support

Across various hospital settings, timings, and types of organ, the transplant experiences reported by our participants share four common "key moments": (i) being notified that they will need to undergo an organ transplant; (ii) receiving the call once an organ is available; (iii) the immediate post-transplant period; and (iv) becoming aware of one's mortality. Table 3 presents interviews excerpts.

3. Previous experiences of sharing self-narratives

Most participants had experiences of sharing their stories prior to attending our creative workshops, in either spoken form (e.g. in other groups or for an organization) or written form (e.g. a letter to their donor or a web platform). Participants reported that the content of their previous self-narratives focused on advice and encouragements, resulting in valorization of experiential knowledge. Most participants felt that their previous experiences of sharing stories had been beneficial to their audience, namely by making others feel less isolated in their journey. However, two participants indicated that previous experiences of sharing self-narratives had been emotionally difficult, due to the act of opening up to others and being confronted by their immediate reactions.

For peer review only

For peer review only - http://bmjopen.bmj.com/site/about/guidelines.xhtml

Most participants also had previous experiences of hearing other transplant patients' stories, in either spoken form (e.g., in other support groups or in the hospital setting) or written form (e.g., a web platform). Participants highlighted the following needs that were met through hearing others' self-narratives: i) access to experiential knowledge different from healthcare professionals' expertise; and ii) encouragement, hope and reassurance. However, it is worth noting that one participant reported feeling overwhelmed and "saturated" after bearing witness to "too many narratives". Table 4 presents interview excerpts.

Experiences of attending creative writing workshop

1. Participant characteristics

Nine lung transplant recipients, four liver transplant recipients and one heart transplant recipient took part in 17 individual interviews to document their experience of attending creative writing workshops. Three participants completed two interviews after attending different creative writing workshops. Eight of them were female and six were male. They were in their fifties and 13 were White. The majority had a post-secondary education level. The average time since the transplant was more than seven years. Table 2 summarizes the participants' characteristics.

2. Positive and negative aspects of the creative writing workshops

Participants enjoyed the creative writing workshops. They reported that the creative writing workshops provided a respectful environment, with encouraging, open-minded, and qualified facilitators. Many participants conveyed that the creative writing workshops allowed them to reflect and gain better insight on their transplantation journey. The group setting was also reported as a notable strength of the workshops, providing a space for peer support and a feeling of connection to others. Many participants also highlighted that the workshops were stimulating and inspired them to invest in their creative process. Importantly, several participants reported that their creative writings had a positive effect on them by

For peer review only - http://bmjopen.bmj.com/site/about/guidelines.xhtml

enabling self-expression and, more specifically, emotional expression. Some participants also mentioned that the workshops strengthened their understanding and appreciation of organ donation. Of note, one participant who had undergone organ transplant several years prior to workshops emphasized that their primary interest in attending the workshops at the present time was to have a space for creativity that remains separate from their transplantation experience per se. Similarly, another participant indicated that they were attracted by the "opportunity to break free from the 'sick talk' and to instead delve into the arts". Most participants would recommend the creative workshops to others who have undergone or are awaiting an organ transplant procedure.

The potential benefits of the creative writing workshops were clearly recognized by most participants. There was, however, a noteworthy heterogeneity regarding the participants' experiences of the creative aspect of the workshops. While some participants reported feeling comfortable with the framework of the creative workshops, several of them reported feeling underqualified and intimidated by others' artistic talents, with two participants describing a competitive environment among participants. Six participants expected the workshops to focus on the organ transplant experience specifically, and so they were disappointed by their perceived lack of testimonials through others' artworks. In terms of logistics, participants commented on the length of the sessions and on the virtual setting. Two participants expressed that they would have liked more time for the group to share their creative writings, but one maintained that the sessions felt "lengthy" enough as they were. Moreover, some participants commented on the barriers to fluid communication with other participants when using a virtual platform such as Zoom: for example, the lack of nonverbal cues, the lack of eye contact, and the technical difficulties. This may at least partly account for the five participants who had the impression that others took either too much or too little space during the workshops, which made them feel less comfortable than they would have liked. Table 5 presents interview excerpts.

For peer review only - http://bmjopen.bmj.com/site/about/guidelines.xhtml

3. Therapeutic aspects of creative writing workshops and the associated peer support

The most frequently reported benefit of the creative process was its "therapeutic" effect, especially through the meaning-making that results from conceiving and sharing their creative writing. Almost half of the participants reported being pleased to discover and experiment with new ways of creating during the workshops, which resulted for some in the exploration of a different perspective of their own selves. Several participants also stressed that the creative format of the workshops was crucial to the voicing of their self-narratives and associated emotions. The elaboration and subsequent sharing of their self-narratives made participants feel "proud", "listened to", and "valued as a human being". Some participants also understood the creation of their artworks as a way to pay tribute and to value every stakeholder involved in the process of organ donation.

Participants who attended the creative writing workshops also reported "feeling connected to others". Workshops were viewed as a way to break isolation and as a source of peer support. Several participants noticed that the positive experience of sharing their creative writings with other participants during the workshops empowered them to be vulnerable in a similar way with their spouse or family members. This feeling of belonging within a group or a community was the cornerstone of various other benefits, such as sharing of experiential knowledge, being touched by others' narratives, putting into perspective and normalizing the participants' experiences, modelling resilience among participants, and cultivating open-mindedness and care.

While most of the participants' feedback highlighted beneficial aspects of peer support, some participants acknowledged that to bear witness to testimonials that were at times "darker" could be difficult, or even "triggering". Two participants noted the importance of "detachment" to avoid feeling overly distressed by others' testimonials. Yet, one participant reported "detachment" as an unwanted feeling which emerged upon discerning self-pity in others' testimonials. Table 6 presents interview excerpts.

For peer review only - http://bmjopen.bmj.com/site/about/guidelines.xhtml

For peer review only

For peer review only - http://bmjopen.bmj.com/site/about/guidelines.xhtml

DISCUSSION

This is the first study to examine heart, lung and liver transplant recipients' perspectives on the impact of creative writing workshops on their transplantation journey. We have previously reported the perspectives of kidney transplant candidates, kidney transplant recipients, and living donors on creative writing workshops.[15] However, heart, liver and lung transplant recipients face different challenges than kidney patients do, since transplantation is a life-saving treatment for them—there is no other option, in the way that dialysis represents an option for patients with end-stage kidney disease. Heart, liver and lung transplant candidates have to face the possibility of their mortality if a transplant is not offered, and also if there are serious adverse effects after transplantation. Becoming aware of one's own mortality was a theme reported by participants in our current study, whereas it was not reported by the kidney transplant candidates and kidney transplant recipients in our earlier study.

As reported by kidney transplant patients, the call for a transplant was a key moment in their transplantation journey. In recent studies conducted with heart and lung transplant patients, the call for transplant was associated with fear and anxiety.[24 25] The participants in our study also highlighted the immediate post-transplantation journey. Such experience aligns with the dominant narrative of transplantation, where the transplantation itself is a healing event. This is a linear and restorative narrative where transplantation will allow patients to return to health and have a second chance.[24 26]

For the participants in our study, sharing stories or hearing other patients' stories during their transplantation journey were described as valuable peer support, and were viewed as a way to normalize their own experiences and put them into perspective. Also, sharing the same experiences of organ transplantation could make the listener more empathic and understanding than someone who has not gone through the transplant experience.[27] Moreover, sharing stories and listening to other patients' stories

For peer review only - http://bmjopen.bmj.com/site/about/guidelines.xhtml

could help to better understand their identities as transplant patients but also as an individual. In this sense, peer support is a way of acquiring experiential knowledge.[28] In a previous study, it was even stated that only someone who has gone through the process could understand what heart transplantation is.[29] However, some patients did not want to be exposed to other patients' stories because they believed that each patient's experience is unique and so did not want to be scared by other patients' bad experiences. This aligns with the results of other studies showing that peer support could be valuable for people awaiting a heart or heart/lung transplantation because peers could provide hope, first-hand information and reassurance. However, it was also reported that some patients did not want to hear about other patients' stories because they did not want be scared or anxious about the future. [25]

Previous studies have also examined the role of illness narratives and storytelling that create sense and coherence in the illness experience and serve as a tool for coping.[30 31] Moreover, when patients share their stories with an audience, the act of storytelling lets them negotiate the meanings of the stories and redefine their identities.[27] A recent study conducted with liver transplant recipients used the creation of short videos and writing exercises during workshops in order to develop a digital story.[32] These workshops allow patients to expand their narrative possibilities and go beyond the archetypes associated with organ transplantation. Sharing the digital stories and commenting on other digital stories and videos allow participants in these workshops to create a sense of community and belonging.

For many participants, the creative writing workshops were described as "therapeutic" and made them feel valued as holistic human beings. It was beyond the scope of this research project to assess the therapeutic aspect of creative writing workshops on the care trajectory of participants. However, this incidental reported effect could be explained partly by the peer support and partly by the fact that workshops allowed vulnerable patients to break their isolation during the anxiety-inducing period of the pandemics. That being said, the therapeutic quality of writing, for both mental and physical health, has

For peer review only - http://bmjopen.bmj.com/site/about/guidelines.xhtml

been described previously by Pennebaker.[33] He developed a technique of expressive writing that included the following principles: i) the writer openly acknowledges and accepts their emotions; ii) they become able to express those emotions; iii) they construct a meaningful story; and iv) writing provides a space for increased self-reflection.[34] Given the standardized frame of expressive writing, it is distinct from creative writing where participants are encouraged to use different literary styles and use fiction and imagination in their writings. It is nonetheless striking to note the resemblance between the therapeutic elements described by our own participants and those reported by Pennebaker. A possible explanation is that expressive writing could be understood as a form of creative writing, hence resulting in similar cognitive and emotional processes being activated and similar therapeutic outcomes. A randomized controlled trial comparing expressive writing to neutral writing found peer support to be beneficial post-transplant only if there had been previous self-reflection and emotional expression.[35] We may therefore hypothesize that emotional expression during our creative workshops was central to their potentially therapeutic yield.

Advances in the field of organ transplantation over the past decades have resulted in considerable improvement in post-transplant survival rates.[36] However, organ transplantation is associated with existential challenges such as disruption in the sense of identity or selfhood. This disruption could be related to the alienation related to the disease leading to transplantation and the post-transplant care; to the symbolic nature of the transplanted organ, such as the heart; or to the bodily disruption, and the feeling of interconnectedness with the person who donated the organs.[37 38] Sharing narratives and peer support between transplant recipients was viewed by the participants as a way to normalize the transplantation journey and the changes of identity that could occur.

Finally, transplant programs are increasingly seeking ways to support recipients in the post-transplant period.[39] Research consistently shows that both physical and emotional health in transplant patients are

For peer review only - http://bmjopen.bmj.com/site/about/guidelines.xhtml

closely linked to social support and physical in activities, before and after transplantation.[40] While many studies have highlighted the benefits of individual or group sports for transplant patients, physical activities may not suit every patient's lifestyle or preferences.[41 42] Transplant programs could therefore explore alternative avenues for patient expression and social connection, such as ABI workshops. Like other group-based interventions, ABI workshops can foster (i) the sharing of knowledge and experiences within a community, and (ii) access to guidance and support.[43] As such, they may contribute to long-term self-management among transplant patients.[44] Moreover, these workshops are relatively low-cost and easy to implement, making them a promising option for new initiatives aimed at improving post-transplant quality of life and outcomes.

For peer review only

Limitations

There are some limitations related to our study sample. All our participants came from two urban transplant centres in Quebec. These participants, both before and after the creative writing workshops, were mainly of White ethnicity with a college or university degree, and a mean age of approximately 60 years. Because the creative writing workshops were conducted in French, we were not able to reach out to a greater diversity of patient ethnicities with a lower level of French literacy. The timing of the workshops, which were at night, could have been a barrier for some participants. The required time commitment may suit older, retired participants rather than young, working adults. The virtual format of the workshops, delivered over Zoom during the pandemic, also favoured patients with a stable or available Internet connection and digital literacy.

Another limitation in our data collection arises from the specificity of the multiple processes involved in the creative writing workshops. Although we were attentive to the reported experience of the creative writing process itself, most of the time the participants' answers did not permit a clear distinction between the action of writing and that of sharing one's writing and self-narratives in the workshop; the two actions were confounded together. Further studies are needed to tease out these different processes in relation to their reported therapeutic effects.

For peer review only

Conclusion

In conclusion, our creative workshops provided a space dedicated to individual creative expression as well as the sharing of self-narratives and peer support. Our participants described the creative process as therapeutic, possibly as a result of increased insight, meaning-making, emotional expression, and the feeling of being valued as a human being. Sharing of self-narratives and peer support were also found to be beneficial due to the feeling of connection with others, the sharing of experiential knowledge, and the normalization of experiences. Creative workshops that combine creative self-expression and peer support were found to be a feasible and beneficial intervention among transplant patients. More research is needed for the sake of studying the impacts of creative writing as an arts-based intervention, as opposed to other forms of storytelling: non-artistic (e.g. digital storytelling, life review and reminiscence therapy),[45-47] or psychotherapeutic (e.g. expressive writing).[48-51]

For peer review only

AUTHORSHIP

1. Laurence Ducharme participated in the data analysis and in the writing of the manuscript.
2. Marie-Françoise Malo participated in the conduct of the research and the data analysis, and in the writing of the manuscript.
3. Alyia Affdal participated in the conduct of the research and the data analysis, and in the writing of the manuscript.
4. Fabian Ballesteros participated in the conduct of the research and the data analysis, and in the writing of the manuscript.
5. Laurence Laneuville participated in the data analysis and in the writing of the manuscript.
6. Léonore Brassard participated in the conduct of the research and in the writing of the manuscript.
7. Benjamin Gagnon-Chainey participated in the conduct of the research and in the writing of the manuscript.
8. Pascale Millot participated in the conduct of the research and in the writing of the manuscript.
9. Catherine Mavrikakis participated in the research design, in the conduct of the research and the data analysis, and in the writing of the manuscript.
10. Simon Harel participated in the research design, in the conduct of the research and the data analysis, and in the writing of the manuscript.
11. Marie-Chantal Fortin participated in the research design, in the conduct of the research and the data analysis, and in the writing of the manuscript.

GUARANTOR: Marie-Chantal Fortin (M-C F) is responsible for the overall content as guarantor.

DATA SHARING STATEMENT: Data will be available upon reasonable request to the corresponding author.

For peer review only

TABLE 1. TITLES AND ART MEDIUMS OF THE WORKSHOPS

Title of the Art-Based Intervention	Art Medium
No official title given	Literature
No official title given	Scriptwriting
No official title given	Literature
No official title given	Comics
Creating from Dreams: Transforming the Experience of the Body through Creative Writing	Literature
No official title given	Sound-texts
Memories	Text combined with other art forms (optional)
Memories and poems	Literature (poetry)
No official title given	Sound
L'importance du lieu dans l'écriture.	Literature
The world of zines	Zines
In My Body, There's a Whole World…	Puppetry
Literary and Pictorial Self-Portraiture	Literature
Making a Scene: Writing Everyday Life	Literature

For peer review only

For peer review only - http://bmjopen.bmj.com/site/about/guidelines.xhtml

Table 2. Characteristics of participants in pre-workshop and post-workshop interviews

Characteristics	Pre-worshop[1] N=31 (%)	Post-Workshop N=14 (%)
**Type of organ transplant**		
Lung transplant recipients	22 (71)	9 (64)
Liver transplant recipients	5 (16)	4 (29)
Heart transplant recipients	4 (13)	1 (7)
**Sex**		
Female/male/unknown	17(55) /13 (42) /1 (3)	8 (57)/6 (43)/(0)
**Age (years)**		
Mean ±SD	54.9 +/-12.9<br>Range 29-72	55.1 +/-15.9<br>Range 26-73
**Race**		
White	27 (87)	13 (93)
Other	2(6)	0 (0)
Prefer not to answer	1(3)	1 (7)
Unknown	1(3)	**0 (0)**
**Employment status**		
Retired	10 (32)	6 (43)
Employed/self-employed	15 (48)	6 (43)
Unemployed	5 (16)	1 (7)
Unknown	1 (3)	1 (7)
**Level of education**		
College or university	21(68)	8 (57)
High school	8 (26)	3 (21)
Elementary school	1 (3)	2 (14)
Unknown	1 (3)	1 (7)
**Annual family income**		
More than $100K	5 (16)	4 (29)
$50K to $99,999	13 (42)	3 (21)
Less than $50K	7 (23)	5 (36)
Prefer not to answer	5 (16)	2 (14)
Unknown	1 (3)	0 (0)
**Time since transplant** (months ±SD)	114.3 +/-78.6<br>Range from 29-396	87.7 +/-57.9<br>**Range from** 9-188

1. Only 30 participants completed the socio-demographic questionnaire

For peer review only - http://bmjopen.bmj.com/site/about/guidelines.xhtml

TABLE 3: KEY MOMENTS IN THE TRANSPLANTATION JOURNEY

**Themes and interview excerpts**	**N=31**
**Being notified that they will need to undergo an organ transplant**<br><br>*"The most shocking moment, well, I think it was the moment when I was told that my liver was no longer good and that I was going to die, and that I was going to be on a waiting list for 6 months."* (male liver recipient)<br><br>*"It was when they told me that it was taking hold of me, that it was really taking hold of my heart. It's gives you...it gives you a bit of a shock. In the end, when you're faced with a fait accompli, the question is whether to let go and die or accept the heart."* (male heart recipient)	**5**
**Receiving the call once an organ is available**<br><br>*"...I got the phone call on the Monday, two days beforehand, saying I had to go in. You're afraid, but at the same time you're so happy, because for me it was just a matter of months then. She told me that it wasn't a matter of years anymore, so we were really looking forward to it."* (female liver recipient)<br><br>*"But it's really true that the call that came that night, well, I was called in the evening then I had to go into the hospital the next morning, but I think it was the evening, I would say it was the worst night of my life because you're saying to yourself, well tomorrow it's make or break, and you are hyper-aware of everything and the hours become very long. At the same time it goes by fast, but at the same time it feels long. You want to be positive and say to yourself, I'm going to come back, I'm going to come back in a few weeks, but at the same time you can't ignore the fact that the risk is there and that these are major surgeries. So I think that that was one of the heaviest experiences in my life."* (female lung recipient)	**5**
**The immediate post-transplant period**<br>*"One moment I remember was when I was all alone in my hospital room and it had been maybe 2 or 3 days, I don't know, a few days since my transplant, and then I was very, very vulnerable emotionally and then I thought about my donor and his family and it brought tears to my eyes. I was, it was mixed emotions because I was so happy, so grateful. I still am but I mean I was so like touched by the gesture, I was so like: Ah my god, it's true, I've got my lungs now. There."* (female lung recipient)<br><br>*"Then after that, there was a kind of euphoria that set in once I'd been transplanted, woken up, everything was fine. In the weeks that followed, I remember I was laughing so hard I couldn't stop, I was in a state of euphoria."* (female lung recipient)	**5**
**Becoming aware of one's mortality**<br>*"There were times when I really thought I was going to die, and now I'm having recurring nightmares. It's been three and a half years now, and it's left a real mark on me, but it's not so much a positive thing as a big change. I don't know."* (female lungs recipient)<br><br>*"Oh my God!" (Hesitations) "There are a few. I think the most memorable moment was one morning when I woke up at home and I was having a pulmonary embolism and thought I was going to die." (Sobbing)* (female lung recipient)	**6**

For peer review only - http://bmjopen.bmj.com/site/about/guidelines.xhtml

TABLE 4 – EXPERIENCES OF SHARING AND HEARING TRANSPLANT SELF-NARRATIVES BEFORE THE WORKSHOPS

Interview excerpts	N=31
**Prior experience of sharing their own stories with other people**	
**1. Self-narratives are a source of advice and information for other patients**	7
"People want to know how it's gonna go, if you have any tips or tricks for them or if there's anything they should know. They want to know about your experience, how things went for you." (female liver recipient)<br>*"I encouraged several others to go through with the transplant. They would say, no, it's too difficult, but I would tell them to take it one day at a time, they didn't have a choice. It's either a transplant or you die. You don't have a choice. You're better off accepting the transplant right off the bat."* (female lung recipient)	
**2. Sharing transplant stories are a way to break the feeling of loneliness**	
"I think they feel less alone because even if you're surrounded by family, sometimes family doesn't truly understand what you're going through." (female liver recipient)	12
**3. Sharing transplant stories could have negative impact**	
"One person told me that I had scared them." (male lung recipient)	3
"I'd say that like the person I'd met face to face with her mother, well I saw that it had helped her a lot, but when I went back home, I had, it was like an emotional burden that was kind of heavy to bear. I'd found it a bit difficult there as it's still quite draining, we can definitely say. I think it's maybe not so for everyone, how can I put it? To open up so much here." (female lung recipient)<br>*"I got angry because I felt that, at least for me, we're all different, and then the person was waiting for a transplant, and then she was, I felt that she was kind of disrespectful toward the transplant request, and then I just stopped talking to her because it just made me angry and then it just made me mad to see that there was like no respect toward the donor afterwards when he had his transplant there."* (female lung recipient)	
**Prior experience of hearing other patients' stories**	
**1. Access to experiential knowledge**	11
"People want to know what's going to happen. People want to know how it's going to be, people want to know do you have stuff, is there anything I should know? They want to know about your experience, how it went for you too." (female liver recipient)<br>*"She told me about her journey, her transplant. She too had a rather difficult transplant, and today she was well, she had a girlfriend and then her girlfriend had a child. So because there was that situation as well, there was a worry if I'm going to be immunosuppressed while living with a child who brings all the germs from daycare. So to have that reference and then she'd say: You just need to wash your hands more. It's going to be all right then."* (female lung recipient)	
**2. Encouragement, hope and reassurance**	
"It encouraged me... We all know that after a transplant, you're a bit worried about the follow-up and what can happen, and when I saw that she was blossoming and that she had	22

For peer review only - http://bmjopen.bmj.com/site/about/guidelines.xhtml

resumed her youthful life, because we were at the same stage of life, yes, it encouraged me." (female lung recipient)<br><br>*"It was very encouraging to see how well she was doing. So that helped me a lot. ...So I'd say what helped me get through it the most was to see... Well maybe to be aware or to meet people who have great stories there, great successes there. And when I say a great success, it's important to be aware that there's a lot of adapting to do afterwards, and then sometimes there are other little problems that arise, but the fact remains that people can resume their activities and continue to live as normal a life as possible, despite certain limitations."* (female lung recipient)<br><br>*"Well, all of their stories, they've all left their mark on me. Each person is different there. It was all positive, really more the hope that was there because there's no hope when you're waiting for lungs. I had a month to live, so I couldn't wait for it to happen. It's fun to see the positive results."* (female lung recipient)	

For peer review only

For peer review only - http://bmjopen.bmj.com/site/about/guidelines.xhtml

TABLE 5: POSITIVE AND NEGATIVE ASPECTS OF THE CREATIVE WRITING WORKSHOPS

Interview excerpts	N=17
**Positive aspects of creative writing workshops**<br><br>*"There were some good discussions and even between the participants, it was very respectful and encouraging."* (female lung recipient)<br><br>*"It's the type of workshop that allows you to share, to learn, to broaden your outlook and get different perspectives on what others have gone through versus what we have gone through ourselves, all while exploring different facets of art. Whether through writing or other media, being able to participate and chat with others and see what they think builds community."* (female lung recipient)<br><br>*"I liked how open and respectful the workshop atmosphere was. I also liked that, in a more general context, it was okay to participate if we wanted to, but also to hold back at times if it felt better. I liked the format: there was good discussion, everyone could participate, be heard, and circle back on what was created."* (female lung recipient)	14
**Negative aspects of the creative writing workshops**<br><br>*"I found it a bit strange that many participants said absolutely nothing and just looked at the zoom. Maybe it's something I'm uncomfortable with because I don't use zoom very often, so yes, it was strange for me to see other participants watching, but saying absolutely nothing."* (female liver recipient)<br><br>*"I don't know why, when we were sharing our creations, I felt like it was such a personal thing. Maybe I'm getting it wrong and I don't want to... but it seemed that others were trying to impress or that there was some kind of contest to see who was the sickest. I don't know how to put it. I don't want to speak badly about anyone, it was just the impression I got, that it was a contest to see who had it the hardest."* (female lung recipient)<br><br>*"I think it's a bit of a shame that during the week when we went over everyone's artwork, at times there were too many of us for everyone to share in the same room, so we had to split into two groups and then we didn't get to see everyone's results. It was a bit sad, because it's interesting to see everyone's artwork."* (female liver recipient)	10

.

For peer review only - http://bmjopen.bmj.com/site/about/guidelines.xhtml

TABLE 6: IMPACT OF THE CREATIVE WRITING WORKSHOPS AND PEER SUPPORT

Interview excerpts	N=31
**1.Therapeutic effect of the creative writing and sharing them**<br>*"Sharing some pretty personal things allowed me to get rid of some feelings of guilt and it was the first time that I had spoken about certain subjects aloud, especially with people I didn't know, and it helped me, because I didn't feel judged."* (female liver recipient)<br><br>*"I think it helps you make sense of what you've gone through. It helps you put it into words, because not everyone has the same vocabulary or can express themselves easily. Sometimes, hearing others talk about something turns on a light bulb inside you and you're like: Aha! That's true, I've never thought about that or considered it quite like that before. So I think that even if it didn't change anything, it helped me to make some sense of the different things that I've been through."* (female lung recipient)	10
**2. Exploration of different perspectives of their own selves**<br>*"I got an interesting writing experience out of it. I'm pretty shy, so writing allowed me to explore certain paths. At the time of the workshops, I was feeling emotional overload, so I closed up. All in all, I'm satisfied with the experience."* (male lung recipient)<br><br>*"I've spent my life in hospitals. I didn't want my illness to define me, but I really started to feel that that's what was happening. I'm more than my illness."* (female lung recipient)	10
**3. Sharing creative writing is a source of pride**<br>*"I think it helped me realize that every workshop helped me step outside of my comfort zone. All the creative writing, which is really not my forte, was a challenge, and that's what I liked about it."* (male lung recipient)	8
**4. Creative writing is a way to give back**<br>*"It's something that's between us and also the fact that we have someone else's organ inside us, we have a part of someone else within us and it's also important to value this person who gave us life, it's thanks to this person who donated their organ or to their family. We're still here, we still exist, so it's really important to pay tribute to them, yes, in a way."* (female liver recipient)<br><br>*"But I always have, not pleasure, but I like to talk about it and I've also done conferences for the Canadian Liver Foundation and then Partenaire Santé, and I find it so extraordinary that they managed to save my life and that I managed to have two transplants with two different livers in the space of 24 hours, that I'm always happy to be able to share that again because I think it's a feat that they accomplished."* (female liver recipient)	2
**5. Creative writing workshops let one feel connected and alleviate loneliness**<br>*"Every story is important, and hearing different stories helps you feel less alone, because transplantees aren't exactly common."* (female liver recipient)<br><br>*"I recognized my own story and it was an eye-opener. We think we're unique, but after hearing those stories, we realize that we really aren't so unique. So, when someone says*	5

For peer review only - http://bmjopen.bmj.com/site/about/guidelines.xhtml

something, I'll say: Ah! It's exactly the same for me. It doesn't bother me not to be so unique, it's actually comforting." (female lung recipient)	
**6. Sharing narratives with other empower patients to be vulnerable with others**<br><br>*"Well, I was very moved by what people were experiencing, and I was also moved by the perception they had of the two artworks I presented, and they understood the emotions I was conveying through them.... And then it was time to write the text, and I must admit that all the emotions came out at that point. ...because it took me three times because my voice was shaking and then I was very, very moved to tell my story."* (female liver recipient)<br><br>*"It made me realize that I wasn't the only one in much the same situation, and that we'd all been through difficult things for most of us, and that we're all still here, still seeing our doctors, still having our treatments, but it's like we're going, we're not giving up. It's hard to see that there are all kinds of different situations, but they're all difficult for everyone, depending on their circumstances. That's it, I think it made me realize that a lot. "* (female liver recipient)	7
**7. Darker side of hearing other narratives**<br>*"Sometimes it can be upsetting to meet others who, like us, are sick and have the same chronic illnesses and challenges. The whole mental health approach kind of surprises me. I'm quite shocked because there are some pretty depressed people in the group, even though it's a different approach."* (female liver recipient)	3

For peer review only

REFERENCES

1. Beilby S, Moss-Morris R, Painter L. Quality of life before and after heart, lung and liver transplantation. *N Z Med J* 2003;116(1171):U381. [published Online First: 20030404]
2. Cierpka A, Małek K, Horodeńska M. What does it mean to live after heart transplantation? The lived experience of heart transplant recipients. A qualitative study. *Health Psychology Report* 2015;3(2) doi: 10.5114/hpr.2015.49678
3. Anderson SM, Wray J, Ralph A, et al. Experiences of adolescent lung transplant recipients: A qualitative study. *Pediatr Transplant* 2017;21(3) doi: 10.1111/petr.12878 [published Online First: 2017/02/02]
4. Duffy JP, Kao K, Ko CY, et al. Long-term patient outcome and quality of life after liver transplantation: analysis of 20-year survivors. *Ann Surg* 2010;252(4):652-61. doi: 10.1097/SLA.0b013e3181f5f23a
5. Dew MA, DiMartini AF. Psychological disorders and distress after adult cardiothoracic transplantation. *J Cardiovasc Nurs* 2005;20(5 Suppl):S51-66. doi: 10.1097/00005082-200509001-00007
6. Rosenberger EM, DiMartini AF, DeVito Dabbs AJ, et al. Psychiatric Predictors of Long-term Transplant-Related Outcomes in Lung Transplant Recipients. *Transplantation* 2016;100(1):239-47. doi: 10.1097/tp.0000000000000824
7. Bruce A, Daudt H, Breiddal S. Can Writing and Storytelling Foster Self-care?: A Qualitative Inquiry Into Facilitated Dinners. *J Hosp Palliat Nurs* 2018;20(6):554-60. doi: 10.1097/njh.0000000000000478
8. Romm KL, Synnes O, Bondevik H. Creative writing as a means to recover from early psychosis-Experiences from a group intervention. *Arts Health* 2022:1-14. doi: 10.1080/17533015.2022.2130379 [published Online First: 20221012]
9. Synnes O, Romm KL, Bondevik H. The poetics of vulnerability: creative writing among young adults in treatment for psychosis in light of Ricoeur's and Kristeva's philosophy of language and subjectivity. *Med Health Care Philos* 2021;24(2):173-87. doi: 10.1007/s11019-020-09998-5 [published Online First: 2021/01/17]
10. Forzoni S, Perez M, Martignetti A, et al. Art therapy with cancer patients during chemotherapy sessions: an analysis of the patients' perception of helpfulness. *Palliat Support Care* 2010;8(1):41-8. doi: 10.1017/s1478951509990691 [published Online First: 20100218]
11. Kelly MP, Dickinson H. The Narrative Self in Autobiographical Accounts of Illness. *The Sociological Review* 1997;45(2)
12. Currier H, Zimmerman CT. Use of Creative Writing to Illustrate Lived Experiences in Hemodialysis-Dependent Children with Chronic Kidney Failure. *Nephrol Nurs J* 2019;46(3):293-336.
13. Marini MG. Narrative Medicine: Bridging the Gap between Evidence-Based Care and Medical Humanities: Springer International Publishing 2016.
14. Ho EW, Murillo AL, Davis LA, et al. Findings of living donation experiences shared on a digital storytelling platform: A thematic analysis. *PEC Innov* 2022;1:100023. doi: 10.1016/j.pecinn.2022.100023 [published Online First: 20220217]
15. Laneuville L, Ballesteros Gallego F, Affdal A, et al. Perspectives of Kidney Transplant Recipients, Transplant Candidates and Living Kidney Donors on the Role of Patients' Self-narratives and Experiences of Creative Writing Workshops: A Qualitative Study. *Canadian Journal of Kidney Health and Disease* 2022;9:1-17. doi: 10.1177/20543581221132742

For peer review only - http://bmjopen.bmj.com/site/about/guidelines.xhtml

16. Tong A, Sainsbury P, Craig J. Consolidated criteria for reporting qualitative research (COREQ): a 32-item checklist for interviews and focus groups. *Int J Qual Health Care* 2007;19(6):349-57. doi: 10.1093/intqhc/mzm042 [published Online First: 20070914]
17. Declaration of Istanbul Custodian Group. The Declaration of Istanbul on Organ Trafficking and Tranpslant Tourism 2025 [Available from: https://www.declarationofistanbul.org/the-declaration accessed September 15.
18. Hoffmann TC, Glasziou PP, Boutron I, et al. Better reporting of interventions: template for intervention description and replication (TIDieR) checklist and guide. *Bmj* 2014;348:g1687. doi: 10.1136/bmj.g1687 [published Online First: 2014/03/13]
19. Stuckey HL, Nobel J. The connection between art, healing, and public health: a review of current literature. *Am J Public Health* 2010;100(2):254-63. doi: 10.2105/ajph.2008.156497 [published Online First: 2009/12/19]
20. Sandelowski M. What's in a name? Qualitative description revisited. *Res Nurs Health* 2010;33(1):77-84. doi: 10.1002/nur.20362
21. Green J, Thorogood N. Qualitative methods for health research. Third Ed ed. London: SAGE Publiications Ltd 2014.
22. Sandelowski M. Whatever happened to qualitative description? *Res Nurs Health* 2000;23(4):334-40. doi: 10.1002/1098-240x(200008)23:4<334::aid-nur9>3.0.co;2-g
23. Green J, Thorogood N. Qualitative Methods for Health Resaerch. Fourth Edition ed. Thousand Oaks, California: Sage publishing 2018.
24. Mestres-Soler O, Gómez-Ibáñez R, Zuriguel-Pérez E, et al. Expectations of patients awaiting lung transplantation: A qualitative study. *Nurs Open* 2023;10(6):3766-73. doi: 10.1002/nop2.1634 [published Online First: 2023/02/05]
25. Stubber C, Kirkman M. The experiences of adult heart, lung, and heart-lung transplantation recipients: A systematic review of qualitative research evidence. *PLoS One* 2020;15(11):e0241570. doi: 10.1371/journal.pone.0241570 [published Online First: 2020/11/12]
26. Wasson S. Waiting, strange: transplant recipient experience, medical time and queer/crip temporalities. *Med Humanit* 2021;47(4):447-55. doi: 10.1136/medhum-2021-012141 [published Online First: 2021/05/30]
27. Hsieh E. Stories in Action and the Dialogic Management of Identities: Storytelling in Transplant Support Group Meetings. *Research on Language and Social Interaction* 2004;37:39-70.
28. Nilsson K, Westas M, Andersson G, et al. Waiting for kidney transplantation from deceased donors: Experiences and support needs during the waiting time -A qualitative study. *Patient Educ Couns* 2022;105(7):2422-28. doi: 10.1016/j.pec.2022.02.016 [published Online First: 2022/03/12]
29. Mauthner O, De Luca E, Poole J, et al. Preparation and Support of Patients through the Transplant Process: Understanding the Recipients' Perspectives. *Nurs Res Pract* 2012;2012:547312. doi: 10.1155/2012/547312 [published Online First: 2012/11/06]
30. Lanis A, Tu E, Peskin M, et al. Storytelling of Young Adults with Chronic Rheumatologic Illnesses: A Pilot Study. *Healthcare (Basel)* 2022;10(10) doi: 10.3390/healthcare10101979 [published Online First: 2022/10/28]
31. Rushforth A, Ladds E, Wieringa S, et al. Long Covid - The illness narratives. *Soc Sci Med* 2021;286:114326. doi: 10.1016/j.socscimed.2021.114326 [published Online First: 2021/08/24]
32. Wong-Mersereau C, Best FA, Tarachansky L, et al. Layered Methodologies: Innovating Multimodal Qualitative Research in Liver Transplantation. *Journal of Liver Transplantation* 2023;12:1-10.
33. Pennebaker J. "Telling stories: the health benefits of narrative". *Lit Med* 2000;19(1):3-18.

For peer review only

34. Ruini C, Mortara CC. Writing Technique Across Psychotherapies-From Traditional Expressive Writing to New Positive Psychology Interventions: A Narrative Review. *J Contemp Psychother* 2022;52(1):23-34. doi: 10.1007/s10879-021-09520-9 [published Online First: 20210914]
35. Rini C, Austin J, Wu LM, et al. Harnessing benefits of helping others: a randomized controlled trial testing expressive helping to address survivorship problems after hematopoietic stem cell transplant. *Health Psychol* 2014;33(12):1541-51. doi: 10.1037/hea0000024 [published Online First: 20131125]
36. Meyer KC. Recent advances in lung transplantation. *F1000Res* 2018;7 doi: 10.12688/f1000research.15393.1 [published Online First: 20181023]
37. Mauthner OE, De Luca E, Poole JM, et al. Heart transplants: Identity disruption, bodily integrity and interconnectedness. *Health (London)* 2015;19(6):578-94. doi: 10.1177/1363459314560067 [published Online First: 2014/12/03]
38. Svenaeus F. Organ transplantation and personal identity: how does loss and change of organs affect the self? *J Med Philos* 2012;37(2):139-58.
39. Kidney Foundation of Canada. Improving Egnagement and Empowering Patients on their Transplant Journey 2022 [Available from: https://kidney.ca/KFOC/media/images/PDFs/PAAC-Project_Final-Report-EN.pdf accessed November 3 2025.
40. Dunn MA, Rogal SS, Duarte-Rojo A, et al. Physical Function, Physical Activity, and Quality of Life After Liver Transplantation. *Liver Transpl* 2020;26(5):702-08. doi: 10.1002/lt.25742 [published Online First: 2020/03/05]
41. Burra P, De Bona M. Quality of life following organ transplantation. *Transpl Int* 2007;20(5):397-409. doi: 10.1111/j.1432-2277.2006.00440.x [published Online First: 2007/04/04]
42. Malo MF, Janaudis-Ferreira T, Affdal A, et al. KEeP ACTIVe Club Study: Kidney Transplant Recipients' Experiences of a Physical Activity and Social Interaction Virtual Group. *Can J Kidney Health Dis* 2024;11:20543581241229254. doi: 10.1177/20543581241229254 [published Online First: 2024/02/12]
43. Vassilev I, Rogers A, Kennedy A, et al. The influence of social networks on self-management support: a metasynthesis. *BMC Public Health* 2014;14:719. doi: 10.1186/1471-2458-14-719 [published Online First: 2014/07/16]
44. Rogers A, Vassilev I, Sanders C, et al. Social networks, work and network-based resources for the management of long-term conditions: a framework and study protocol for developing self-care support. *Implement Sci* 2011;6:56. doi: 10.1186/1748-5908-6-56 [published Online First: 2011/05/31]
45. Wood EH, Waterman AD, Pines R. Storytelling to Inspire Dialysis Patients to Learn about Living Donor Kidney Transplant. *Blood Purif* 2021;50(4-5):655-61. doi: 10.1159/000512651 [published Online First: 2021/01/14]
46. Rios Rincon AM, Miguel Cruz A, Daum C, et al. Digital Storytelling in Older Adults With Typical Aging, and With Mild Cognitive Impairment or Dementia: A Systematic Literature Review. *J Appl Gerontol* 2022;41(3):867-80. doi: 10.1177/07334648211015456 [published Online First: 2021/05/20]
47. King KD. Bringing Creative Writing Instruction into Reminiscence Group Treatment. *Clin Gerontol* 2018;41(5):438-44. doi: 10.1080/07317115.2017.1405858 [published Online First: 2017/12/20]
48. Blechinger T, Klosinski G. [The meaning of bibliotherapy and expressive writing in child and adolescent psychiatry]. *Prax Kinderpsychol Kinderpsychiatr* 2011;60(2):109-24. doi: 10.13109/prkk.2011.60.2.109 [published Online First: 2011/03/24]
49. Costa A, Abreu M. Expressive and creative writing in the therapeutic context: From the different concepts to the development of writing therapy programs. *Psychologica* 2018;61(1):69-86.

For peer review only - http://bmjopen.bmj.com/site/about/guidelines.xhtml

50. Pennebaker JW. Telling stories: the health benefits of narrative. *Lit Med* 2000;19(1):3-18. doi: 10.1353/lm.2000.0011 [published Online First: 2000/05/29]
51. Smyth JM, Pennebaker JW. Exploring the boundary conditions of expressive writing: In search of the right recipe. *Br J Health Psychol* 2008;13(Pt 1):1-7. doi: 10.1348/135910707X260117 [published Online First: 2007/11/24]

For peer review only

For peer review only - http://bmjopen.bmj.com/site/about/guidelines.xhtml

Figure 1 – Flowchart of the Recruitment Process

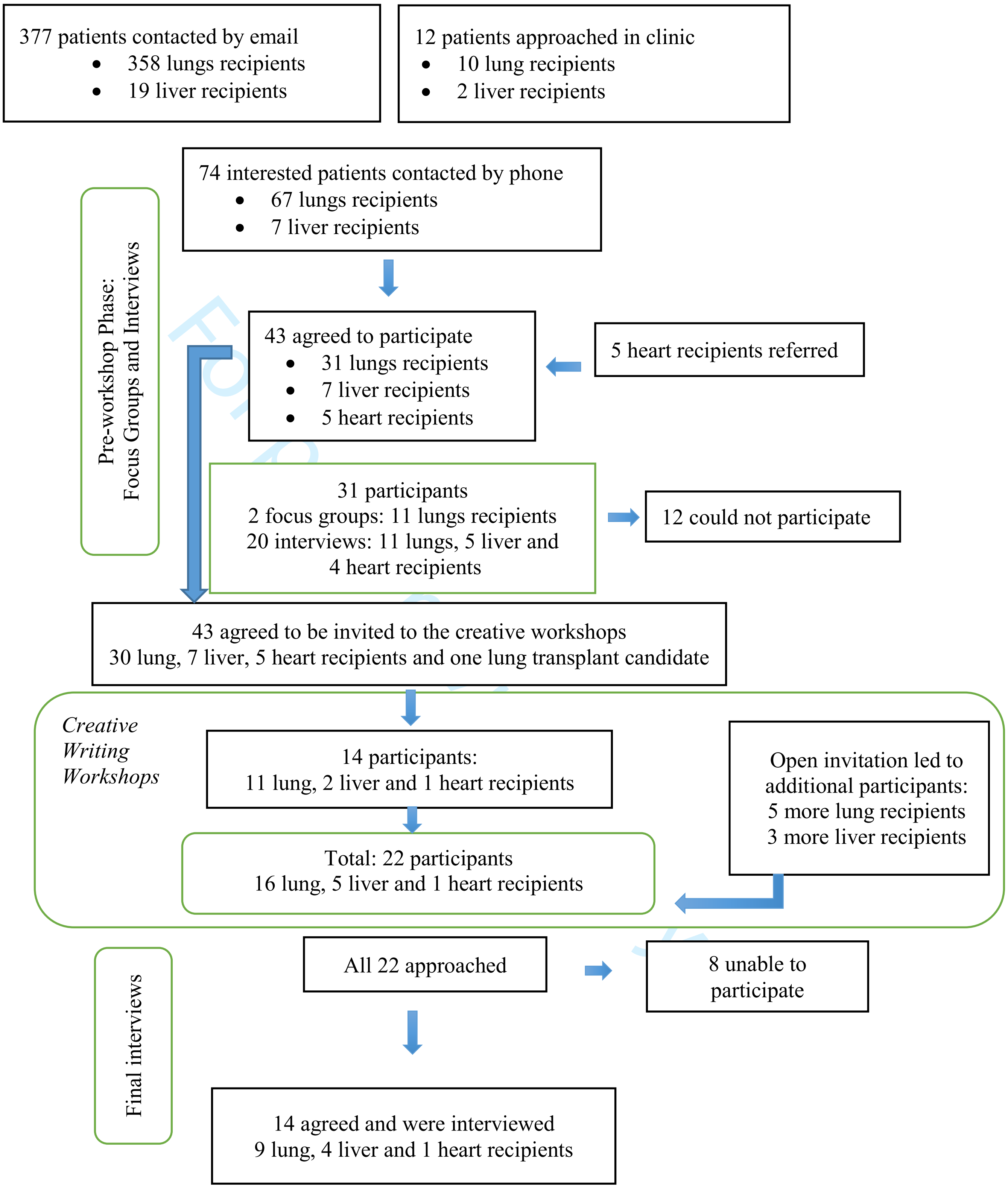

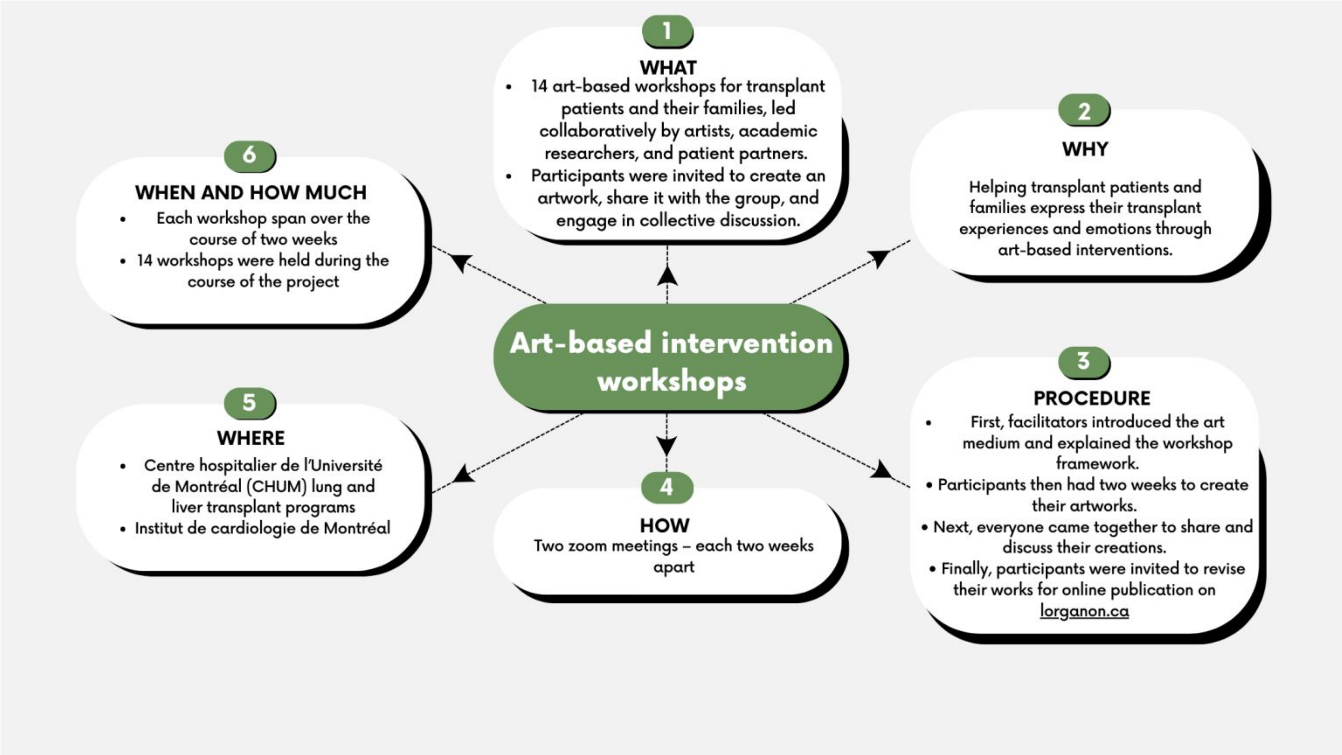

Figure 2 - Description of the art-based intervention using the TIDieR checklist

SUPPLEMENTARY MATERIAL:

PRE-WORKSHOP FOCUS GROUP AND INTERVIEW QUESTION GUIDE:

1. What is the most significant moment in your transplant story?
2. Have you ever shared your story with other patients?
   a. If yes:
   i. What did you share? In what context?
   ii. What impact did this story have on the other person? On you?
   b. If not, why?
3. What is your experience of receiving stories from transplanted patients?
   a. At what point did you receive these stories?
   b. What was the impact of these stories on your journey?
   c. What did you appreciate about these stories?
4. In your opinion, can patient stories be beneficial?
   a. How?
   b. What elements should be included in these stories?
   c. At what point in the care trajectory?
   i. During pre-transplant evaluation?
   ii. During registration and waiting period?
   iii. During the transplant and immediate post-op?
   iv. The first year post-transplant?
   v. Throughout the transplant journey?
   vi. During graft loss?
   d. Who should be the narrators of these stories?
5. Do you recall transplant stories told through media such as movies, novels, comics, or clips?
   a. What reactions did you have when seeing, reading, or hearing these stories?
6. What do you think about the idea of developing a web platform that showcases the diversity of transplant patient journeys?
7. If we were to develop a web platform with patient stories, what form should these stories take?
8. What do you think about healthcare providers stories? Would they be useful? What should they include?
9. How can patient stories help other patients cope with transplantation?

POST-WORKSHOP INTERVIEW QUESTION GUIDE

1. Which creative writing workshops did you participate in?
2. What has been your experience with the creative writing workshops?
3. Which workshop did you enjoy the most? Why?
4. What did you gain from these workshops?
5. What aspects of these workshops did you like?
6. What aspects did you like less and think could be improved?
7. What has been your experience of sharing your transplant story with others?
8. What impact did the stories of other participants have on you?
9. How do you envision your texts and creations on the web platform?
10. How have these workshops affected you?
11. How have these workshops changed your experience of transplantation or illness?
12. Would you recommend such workshops to someone close to you?
13. Do you have any other comments you would like to share with me?

For peer review only
